# The transcription factor TpRfx1 is an essential regulator of amylase and cellulase gene expression in *Talaromyces pinophilus*

**DOI:** 10.1186/s13068-018-1276-8

**Published:** 2018-10-08

**Authors:** Gui-Yan Liao, Shuai Zhao, Ting Zhang, Cheng-Xi Li, Lu-Sheng Liao, Feng-Fei Zhang, Xue-Mei Luo, Jia-Xun Feng

**Affiliations:** State Key Laboratory for Conservation and Utilization of Subtropical Agro-bioresources, Guangxi Research Center for Microbial and Enzyme Engineering Technology, College of Life Science and Technology, Guangxi University, 100 Daxue Road, Nanning, 530004 Guangxi People’s Republic of China

**Keywords:** *Talaromyces pinophilus*, Transcription factor, Regulation, Amylase, Cellulase

## Abstract

**Background:**

Perfect and low cost of fungal amylolytic and cellulolytic enzymes are prerequisite for the industrialization of plant biomass biorefinergy to biofuels. Genetic engineering of fungal strains based on regulatory network of transcriptional factors (TFs) and their targets is an efficient strategy to achieve the above described aim. *Talaromyces pinophilus* produces integrative amylolytic and cellulolytic enzymes; however, the regulatory mechanism associated with the expression of amylase and cellulase genes in *T. pinophilus* remains unclear. In this study, we screened for and identified novel TFs regulating amylase and/or cellulase gene expression in *T. pinophilus* 1-95 through comparative transcriptomic and genetic analyses.

**Results:**

Comparative analysis of the transcriptomes from *T. pinophilus* 1-95 grown on media in the presence and absence of glucose or soluble starch as the sole carbon source screened 33 candidate TF-encoding genes that regulate amylase gene expression. Thirty of the 33 genes were successfully knocked out in the parental strain *T. pinophilus* ∆*TpKu70*, with seven of the deletion mutants firstly displaying significant changes in amylase production as compared with the parental strain. Among these, ∆*TpRfx1* (*TpRfx1*: *Talaromyces pinophilus Rfx1*) showed the most significant decrease (81.5%) in amylase production, as well as a 57.7% reduction in filter paper cellulase production. Real-time quantitative reverse transcription PCR showed that *TpRfx1* dynamically regulated the expression of major amylase and cellulase genes during cell growth, and in vitro electrophoretic mobility shift assay revealed that TpRfx1 bound the promoter regions of genes encoding α-amylase (*TP04014*/*Amy13A*), glucoamylase (*TP09267*/*Amy15A*), cellobiohydrolase (*TP09412*/*cbh1*), β-glucosidase (*TP05820*/*bgl1),* and endo-β-1,4-glucanase (*TP08514*/*eg1*). TpRfx1 protein containing a regulatory factor X (RFX) DNA-binding domain belongs to RFX family.

**Conclusion:**

We identified a novel RFX protein TpRFX1 that directly regulates the expression of amylase and cellulase genes in *T. pinophilus*, which provides new insights into the regulatory mechanism of fungal amylase and cellulase gene expression.

**Electronic supplementary material:**

The online version of this article (10.1186/s13068-018-1276-8) contains supplementary material, which is available to authorized users.

## Background

The filamentous fungus *Talaromyces pinophilus*, formerly *Penicillium pinophilum* in the *Penicillium* subgenus *Biverticillium*, is a member of the family *Trichocomaceae* belonging to the order *Eurotiales* (class *Eurotiomycetes*; phylum *Ascomycota*) [1]. *T. pinophilus* produces yellow colonies, darker-green conidium, and red pigment, with changes in colony color to yellow, orange, or red-to-purplish red shades on potato dextrose agar (PDA) plates [2].

*Talaromyces pinophilus* has been potentially applied in the biotechnological industry due to its ability to produce integrative amylolytic and cellulolytic enzymes [3]. Amylases, including α-amylase (EC 3.2.1.1), glucoamylase (EC 3.2.1.3), α-glucosidase (EC 3.2.1.20), and 1,4-α-glucan-branching enzyme (EC 2.4.1.18), degrade starches, with α-amylase attacking the α-1,4-glycosidic bonds of amylopectin or amylose to generate varying lengths of straight chains and branched oligosaccharides and glucoamylase breaking α-1,4- or α-1,6-glucosidic linkages at the nonreducing ends of starch chains or dextrin [4].

Cellulases include endo-β-1,4-glucanase (EG; EC 3.2.1.4), cellobiohydrolase (CBH; EC 3.2.1.91), and β-glucosidase (BGL; EC 3.2.1.21), with EG attacking internal β-1,4-glycosidic bonds of cellulose chains to release chain ends, CBH hydrolyzing cellulose chains from both ends to release cellobiose, and BGL hydrolyzing the resulting soluble cellooligosaccharides and cellobiose products into glucose [5].

Transcriptional expression of fungal amylase and cellulase genes is regulated by transcription factors (TFs), with the expression of both enzyme genes co-regulated under certain conditions. A lack of the TF AmyR induces the expression of cellulase genes and represses the transcription of amylase genes in *Penicillium oxalicum* [6] and *Aspergillus niger* [7]. Conversely, the high-mobility group box protein PoxHmbB positively regulates the expression of major cellulase genes and negatively controls the expression of amylase genes in *P*. *oxalicum* [8]. Additionally, the deletion of *creA*/*creB* involved in carbon catabolite repression response to glucose not only improves α-amylase production but also enhances cellulase and xylanase activities [9]. However, studies identifying TFs responsible for co-regulating the expression of amylase and cellulase genes are limited and insufficient to describe the regulatory mechanism(s) associated with the expression of fungal enzyme genes involved in plant biomass degradation.

Regulatory factor X (RFX) family proteins regulate both cellular differentiation and the cell cycle [10] and contain an RFX DNA-binding domain belonging to the winged-helix subfamily of helix-turn-helix proteins [11]. Since the identification of RFX1 in mammals, several conserved members from yeast to humans, as well as filamentous fungi, have been isolated [10, 12, 13], including RTX1-7 in humans [14], Snf1-activating kinase 1 (Sak1) in *Schizosaccharomyces pombe* [15], cephalosporin C regulator 1 (CPCR1) in *Acremonium chrysogenum* [16], RfxA in *Talaromyces marneffei* (formerly *Penicillium marneffei*) [12], and PcRFX1 in *Penicillium rubens* (formerly *Penicillium chrysogenum*) [13]. Among these, RTX1-7 is critical for the development of serious human diseases [14], and Sak1 promotes mitotic exit mediated by cAMP-dependent protein kinase [15]. Moreover, RfxA in *T. marneffei* controls mycelial growth and morphogenesis by regulating cell-division events [12], and PcRFX1 regulates the expression of β-lactam-biosynthesis genes [13]. However, the regulation of RFX proteins in plant biomass-degrading enzyme production in filamentous fungi, including *Talaromyces* spp., remains unknown.

*Talaromyces pinophilus* 1-95 isolated from ploughed soil in China can produce highly active calcium-independent amylase and integrative cellulase [2], both potentially applicable to plant biomass biorefining. Recently, the *T. pinophilus* 1-95 genome was sequenced [3]. In the current study, we screened and identified novel TFs that regulate the expression of amylase and cellulase genes in *T. pinophilus* through comparative transcriptome profiling and genetic analyses. Here, we describe a key regulatory gene, *TpRfx1* (*Talaromyces pinophilus Rfx1, TP06218*), encoding an RFX protein that directly regulates the expression of amylase and cellulase genes.

## Results

### Seven novel TFs regulate amylase production in *T. pinophilus*

To screen candidate genes that regulate amylase production in *T. pinophilus* 1-95, we preformed comparative analyses of transcriptomes from strain 1-95 grown in media in the presence and absence of glucose or soluble starch as the sole carbon source. The results identified 33 candidate genes exhibiting expression levels higher than that of *creA* in strain 1-95 with glucose relative to strain 1-95 in the absence of a carbon source or *amyR* in strain 1-95 with starch relative to strain 1-95 with glucose or in the absence of a carbon source (Additional file 1: Table S1).

**Fig. 1:**
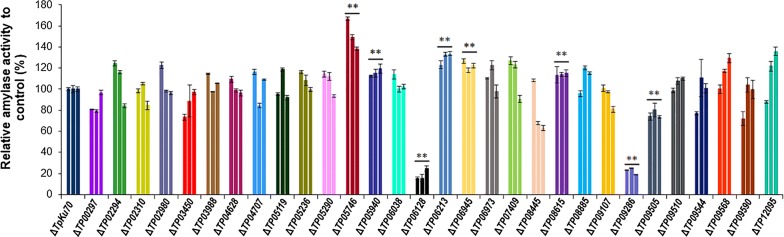
The amylase activities of crude enzymes produced by deletion mutants of the candidate regulatory genes grown with soluble corn starch used as the sole carbon source for 5-days post-inoculation. All experiments were performed independently in triplicate. **P ≤ 0.01 and *P ≤ 0.05 between the deletion mutants and the parental strain ∆*TpKu70* by Student *t* test

The 33 candidate genes were then subjected to knockout experiments following homologous recombination in the parental strain derived from the wild-type 1-95 strain through deletion of *TpKu70*, which is involved in non-homologous end joining [17]. Ultimately, 30 deletion mutants were successfully constructed and validated with PCR (Additional file 2: Figure S1A–W) using specific primers (Additional file 3: Table S2), with a targeting rate of 91%. Measurement of amylase production revealed that eight of the deletion mutants, including the known ∆*TpAmyR*, showed significant changes in amylase production (*P* < 0.05, Student *t* test) ranging from 18.5 to 151.4% of amylase production of ∆*TpKu70* (Fig. 1). Of the genes deleted in these eight mutants, seven genes (*TP05746*, *TP05940*, *TP06128*, *TP06213*, *TP06945*, *TP08615*, and *TP09505*) were here firstly reported to be involved in amylase production in *T*. *pinophilus* (Table 1). Interestingly, ∆*TP06128* showed the 75.4% to 84.5% decrease in amylase activity as compared with ∆*TpKu70* when grown in the presence of soluble starch, suggesting *TP06128* as the most important regulatory gene among the seven genes. Therefore, the gene *TP06128* was chosen for further investigation.

**Table 1:** The found novel regulatory genes in this study regulating amylase production in *T. pinophilus* 1-95

Gene ID	GenBank accession number	InterPro annotation^a^	Domain description
*TP05746*	MH447996	IPR001138	Zinc finger, Zn2Cys6 type
		IPR007219	Fungal_Trans
*TP05940*	MH447994	IPR011991	Winged helix repressor
			DNA-binding domain
*TP06128*	MH447992	IPR003150	RFX
			DNA-binding domain
*TP06213*	MH447995	IPR001138	Zinc finger, Zn2Cys6 type
		IPR007219	Fungal_Trans
*TP06945*	MH447993	IPR001138	Zinc finger, Zn2Cys6 type
		IPR007219	Fungal_Trans
*TP08615*	MH447990	IPR001138	Zinc finger, Zn2Cys6 type
		IPR007219	Fungal_Trans
*TP09505*	MH447991	IPR001138	Zinc finger, Zn2Cys6 type
		IPR007219	Fungal_Trans

^a^*IPR* InterPro database (http://www.ebi.ac.uk/interpro/scan.html)

To exclude the possibility of gene insertion at multiple sites in the ∆*TpKu70* genome by the *TP06128*-knockout cassette, Southern hybridization analysis was performed using specific probes (Additional file 3: Table S2) to ensure accurate products (Additional file 2: Fig. S1X). A complementary strain harboring *TP06128* was subsequently constructed and confirmed with PCR using specific primers (Additional file 4: Figure S2 and Additional file 3: Table S2).

### TP06128 encodes an RFX protein containing an RFX DNA-binding domain

**Fig. 2:**
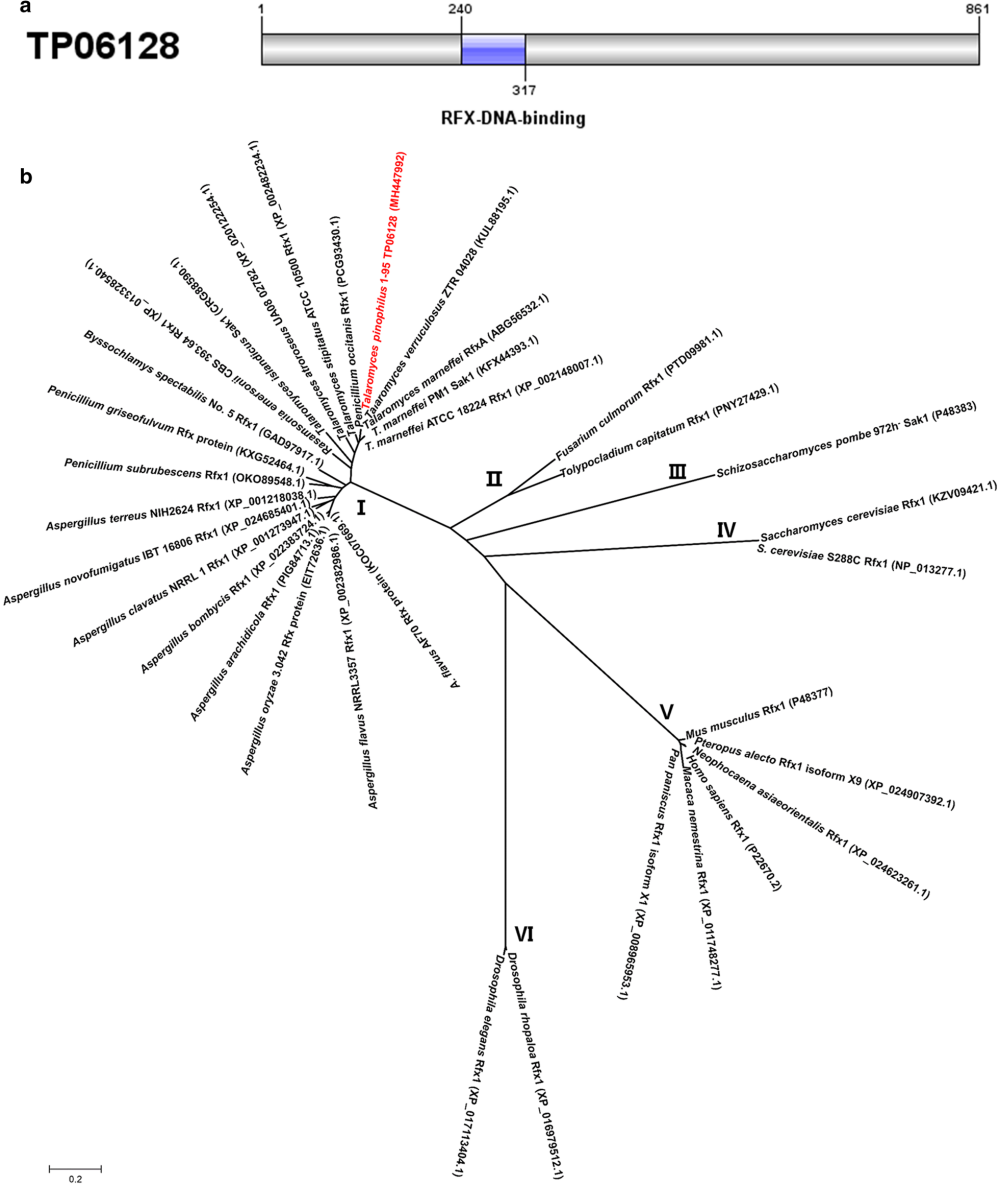
Characterization of TP06128 from *T. pinophilus* 1-95. **a** Conserved-domain analysis using SMART. **b** Unrooted phylogenetic tree for TP06128 and its homologous proteins constructed using MEGA 7.0 based on the neighbor-joining method and a Poisson correction model. Scale bar indicates branch lengths

The *TP06128*-encoded protein comprises 861 amino acids, with SMART analysis (http://smart.embl-heidelberg.de/) revealing the presence of an RFX DNA-binding domain (PF02257; *E* value, 9.1e-28) between residue positions 240–317 (Fig. 2a). Additionally, BlastP (https://blast.ncbi.nlm.nih.gov/Blast.cgi) analysis indicated that the protein TP06128 shares 92% and 27% sequence identity with RfxA from *T*. *marneffei* 2161 (ABG56532.1) and Sak1 from *S. pombe* 972h^−^ (P48383), respectively. TP06128 was subsequently re-designated as TpRfx1 (Tp is the abbreviation of fungal species name *Talaromyces pinophilus*).

Phylogenetic analysis indicated conservation of TpRfx1 (TP06128) and its homologs in filamentous fungi, and specifically in *Talaromyces* sp. The proteins in this tree were divided into six clusters (I–VI), which included those identified in filamentous fungi (I and II), yeast (III and IV), and mammals (V and VI). RFX1 proteins from filamentous fungi were close in evolutionary relationship to those in yeast, followed by those in mammals, and more distant from those in *Drosophila* (Fig. 2b).

### TpRfx1 is required for amylase and cellulase production in *T. pinophilus* under specific induction conditions

**Fig. 3:**
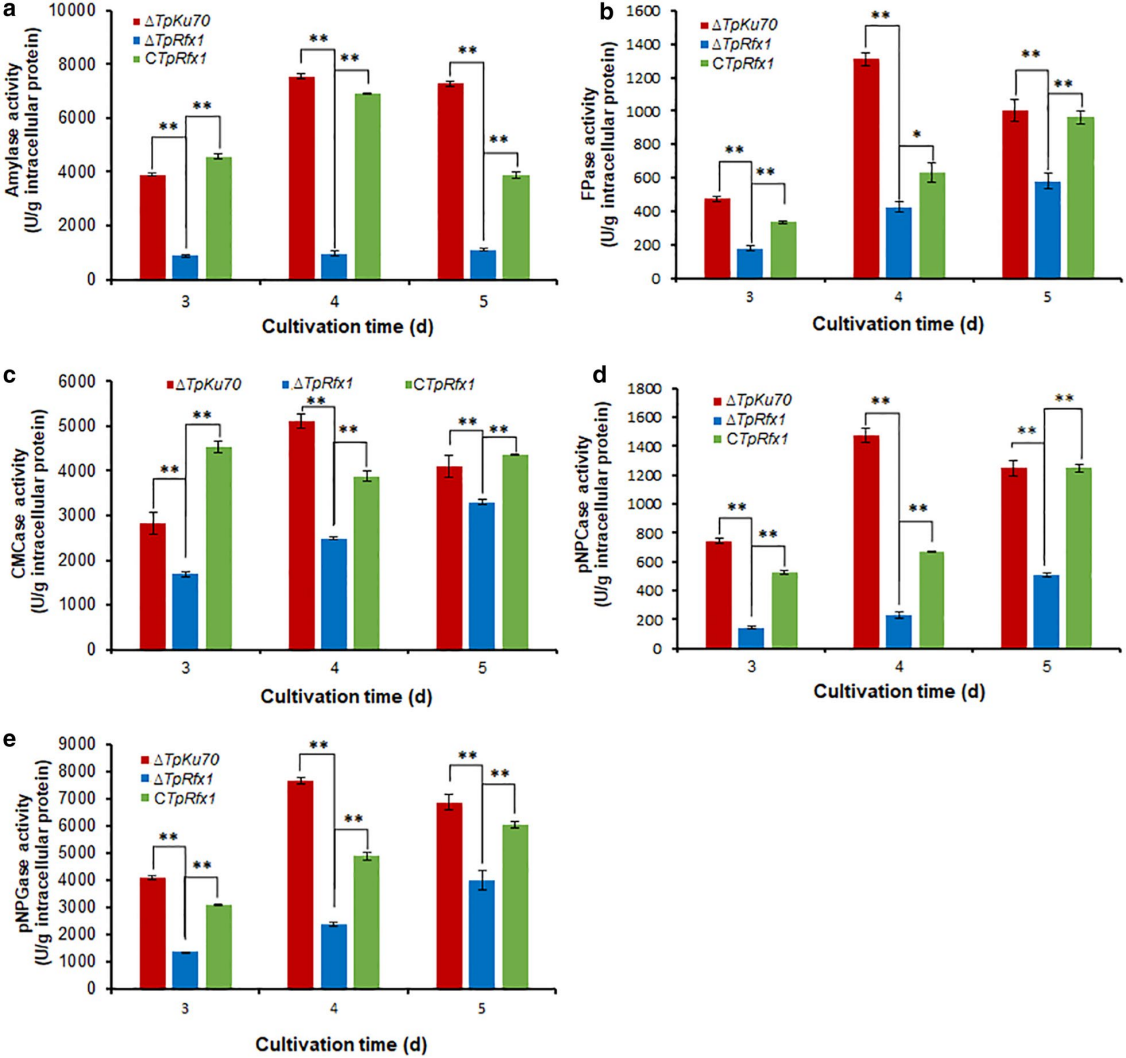
Activities of crude enzymes produced by ∆*TpRfx1*, C*TpRfx1* and ∆*TpKu70*. Crude enzymes were collected from fungal strains grown on SCS or WA for 3–5 days at 28 °C after shifting from glucose. All experiments were performed independently in triplicate. **P < 0.01 and *P < 0.05 between the deletion mutant ∆*TP06128* and the parental strain ∆*TpKu70* or complementary strain C*TpRfx1* by Student *t* test. **a** Amylase activity. **b** Filter paper cellulase (FPase) activity. **c** Carboxy methyl cellulase (CMCase) activity. **d** *p*-nitrophenyl-β-cellobiosidase (pNPCase) activity. **e** *p*-nitrophenyl-β-glucopyranosidase (pNPGase) activity

To investigate the regulatory roles of TpRfx1 in promoting plant biomass degradation by amylases and cellulases in *T. pinophilus*, the ∆*TpRfx1* strain, complementary strain *CTpRfx1*, and parental strain ∆*TpKu70* were transferred into fresh medium containing soluble corn starch (SCS) or wheat bran plus Avicel (WA) for a 5-day culture following a 24-h pre-culture in glucose medium, and their amylase and cellulase activities were measured. The results indicated that ∆*TpRfx1* lost 77.6% to 87.3% amylase activity relative to that observed in ∆*TpKu70* (*P *< 0.01, Student *t* test) under SCS induction, whereas C*TpRfx1* displayed similar amylase activity as that observed in ∆*TpKu70* (Fig. 3a).

Evaluation of cellulase activity showed that the filter paper cellulase (FPase), carboxymethylcellulase (CMCase), *p*-nitrophenyl-β-cellobiosidase (pNPCase), and *p*-nitrophenyl-β-glucopyranosidase (pNPGase) activities of ∆*TpRfx1* were reduced by 42.4% to 67.4%, 19.9% to 40.3%, 59.2% to 84.3%, and 41.7% to 69.0% (*P* < 0.05, Student *t* test), respectively, relative to the activity observed in ∆*TpKu70* under WA induction. As expected, all cellulase activities in C*TpRfx1* were similar to that in the parental strain (Fig. 3b–e).

### TpRfx1 controls *T. pinophilus* sporulation and growth

**Fig. 4:**
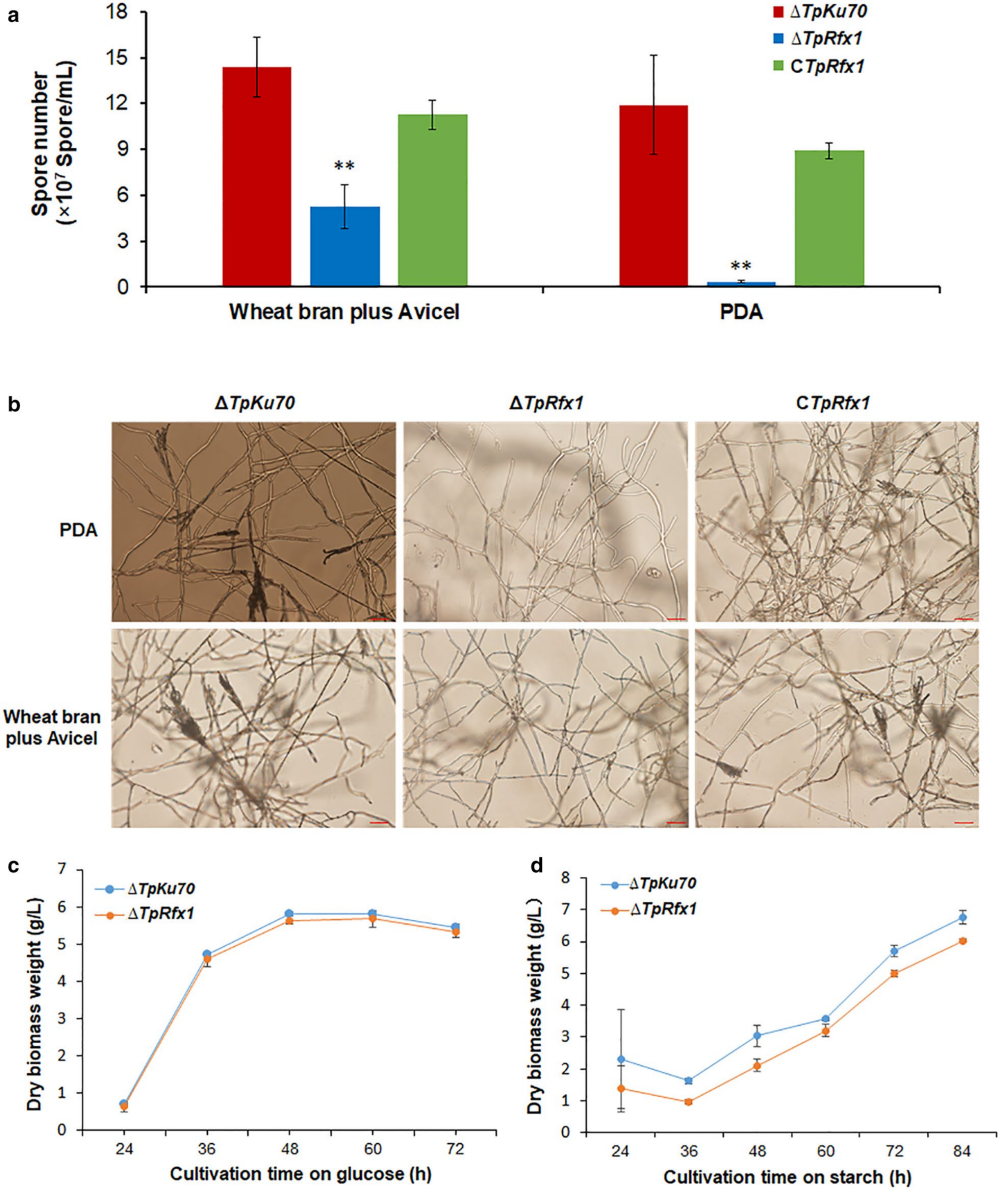
Phenotypic analyses of ∆*TpRfx1*, C*TpRfx1* and ∆*TpKu70*. **a** Quantitative analysis of conidiation on plates containing PDA or WA incubated at 28 °C for 8 days. **P < 0.01 between ∆*TpRfx1* and ∆*TpKu70* by Student *t* test. **b** Microscopic investigation of mycelial sporulation on plates containing PDA or WA. Fungal strains were cultivated at 28 °C for 80 h. **c**, **d** Growth curves of ∆*TpRfx1* and ∆*TpKu70* in medium containing glucose and SCS as the sole carbon source, respectively. In **a**, **c**, **d**, independent experiments were performed in triplicate

To evaluate the effect of *TpRfx1* on *T. pinophilus* phenotype and growth, ∆*TpRfx1*, C*TpRfx1*, and ∆*TpKu70* were incubated on solid plates containing WA as the sole carbon source, with PDA plates used as a control. Asexual spores produced by strains ∆*TpRfx1*, C*TpRfx1*, and ∆*TpKu70* on plates were quantitatively analyzed. The results revealed that ∆*TpRfx1* produced only 36.6% of the spores generated from ∆*TpKu70* when grown on WA plates for 8 days, whereas ∆*TpRfx1* barely produced spores when grown on PDA plates over the same cultivation time. Additionally, C*TpRfx1* exhibited similar levels of spores on both WA and PDA plates as the Δ*TpKu70* (Fig. 4a). Microscopic investigation showed that ∆*TpRfx1* mycelia did not sporulate when grown on both PDA and WA plates for 80 h, whereas ∆*TpKu70* and C*TpRfx1* formed sporangium and produced asexual spores (Fig. 4b).

To determine whether TpRfx1 affects *T. pinophilus* cell growth, growth curves for both ∆*TpRfx1* and ∆*TpKu70* were compared following inoculation into medium containing glucose or SCS as the sole carbon source. ∆*TpRfx1* accumulated similar amounts of mycelial biomass as that by ∆*TpKu70* (Fig. 4c) in glucose medium, whereas the mycelial biomass of ∆*TpRfx1* was slightly lower than that of ∆*TpKu70* in SCS medium (Fig. 4d), suggesting that lack of TpRfx1 affected the utilization of starch by *T. pinophilus*.

### TpRfx1 regulates the expression of amylase and cellulase genes in *T. pinophilus*

**Fig. 5:**
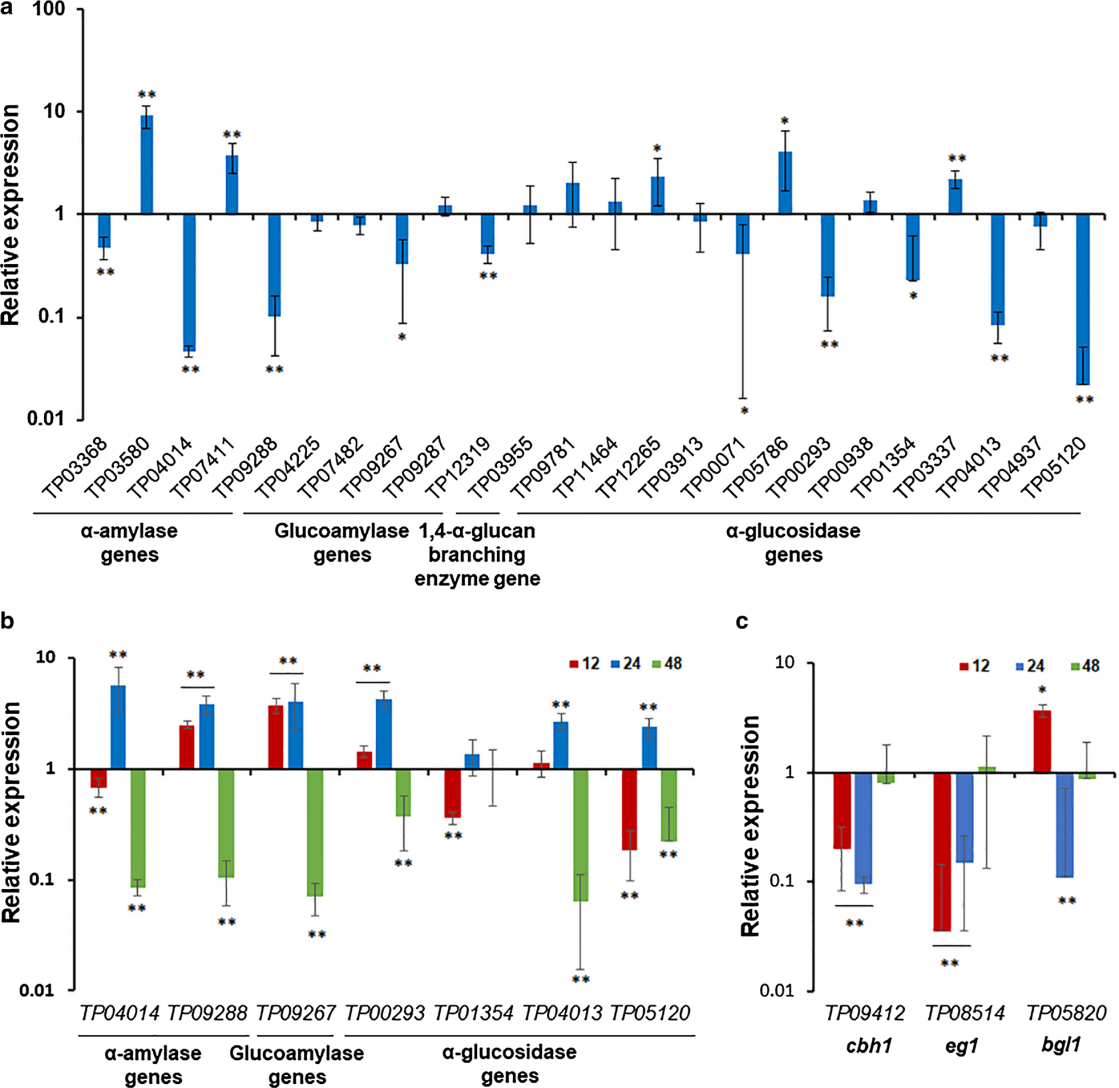
Regulation of amylase and cellulase gene expression by *TpRfx1* in *T. pinophilus*. Expression levels of the tested genes in the ∆*TpRfx1* were normalized against the parental strain ∆*TpKu70*. **P ≤ 0.01 and *P ≤ 0.05 between ∆*TpRfx1* and ∆*TpKu70* by Student *t* test. All experiments were independently performed in triplicate. **a** Expression levels of 24 amylase genes, including five α-amylase genes, five glucoamylase genes, one gene encoding a 1,4-α-glucan-branching enzyme, and 13 α-glucosidase genes. Samples were prepared from fungal strains grown on medium containing SCS for 48 h after shifting from glucose. **b** Expression levels of seven amylase genes, including two α-amylase genes, two glucoamylase genes, and three α-glucosidase genes, under SCS induction. Transcriptional levels were measured by RT-qPCR at three time points (12, 24, and 48 h) after shifting from glucose. **c** Expression levels of three cellulase genes, including one cellobiohydralase gene (*cbh1*), one endo-β-1,4-glucanase gene (*eg1*), and β-1,4-glucosidase gene (*bgl1*), under WA induction. Transcriptional levels were measured with RT-qPCR at three time points (12, 24, and 48 h) after shifting from glucose

To investigate whether TpRfx1 transcriptionally regulates the expression of amylase genes in *T. pinophilus* grown on SCS, real-time quantitative reverse transcription PCR (RT-qPCR) was employed, with ∆*TpKu70* as a control under the same cultivation conditions. The transcription levels of all 24 amylase genes in *T. pinophilus* 1-95 [3], including five α-amylase genes (*TP03368*, *TP03580*, *TP04014*, *TP07411*, and *TP09288*), five glucoamylase genes (*TP04225*, *TP07482*, *TP09267*, *TP09287,* and *TP12319*), 13 α-glucosidase genes (*TP09781*, *TP011464*, *TP12265*, *TP03913*, *TP00071*, *TP05786*, *TP00293*, *TP00938*, *TP01354*, *TP03337*, *TP04013*, *TP04937*, and *TP05120*), and a gene encoding a 1,4-α-glucan-branching enzyme *(TP03955*) were measured at 48 h after shifting from glucose to SCS. The expression of each tested amylase gene in the ∆*TpKu70* as the control was set to 100%. The results showed that the transcripts of 15 genes were significantly altered in ∆*TpRfx1*, including five upregulated by 2.2- to 9.3-fold and 10 downregulated by 52.0% to 97.8% (Fig. 5a), relative to that in the ∆*TpKu70*.

Furthermore, seven amylase genes (*TP04014*, *TP09288*, *TP09267*, *TP00293*, *TP01354*, *TP04013*, and *TP05120*) exhibiting substantial reduction in expression relative to the control were selected to investigate dynamic regulation by TpRfx1. Among these, *TP04014* and *TP09267* were predicted to encode the key α-amylase Amy13A and glucoamylase Amy15A, respectively. Their transcription levels subsequently were measured at 12, 24, and 48 h after the shift from glucose to SCS. The results indicated that only three genes (*TP04014*, *TP01354*, and *TP05120*) exhibited from 31.6 to 81.4% reduced expression in ∆*TpRfx1* at 12 h as compared with that in ∆*TpKu70*. Conversely, *TP09288*, *TP09267*, and *TP00293* showed 1.4- to 3.8-fold elevations in transcript levels in ∆*TpRfx1* at 12 h relative to the control. By contrast, at 24 h, all genes, except *TP01354*, showed upregulated expression ranging from 2.4- to 5.7-fold in ∆*TpRfx1* relative to the control. The transcript levels of all genes were significantly lower in ∆*TpRfx1* as compared with those in ∆*TpKu70* at 48 h (Fig. 5b).

Additionally, we evaluated TpRfx1 regulation of key cellulase genes, including *TP09412* (*cbh1*), *TP08514* (*eg1*), and *TP05820* (*bgl1*), in *T. pinophilus* grown on WA and at 12, 24, and 48 h after the shift from glucose. *TpRfx1* deletion resulted in significant reduction in both *TP09412* and *TP08514* transcripts at 12 and 24 h (*P* < 0.01, Student *t* test), whereas no changes were observed at 48 h. The expression of *TP05820* in ∆*TpRfx1* was increased 3.7-fold at 12 h, decreased 89.1% at 24 h, and the same as levels observed in ∆*TpKu70* at 48 h (Fig. 5c).

### TpRfx1 binds to the promoter regions of genes encoding plant biomass-degrading enzymes in vitro

**Fig. 6:**
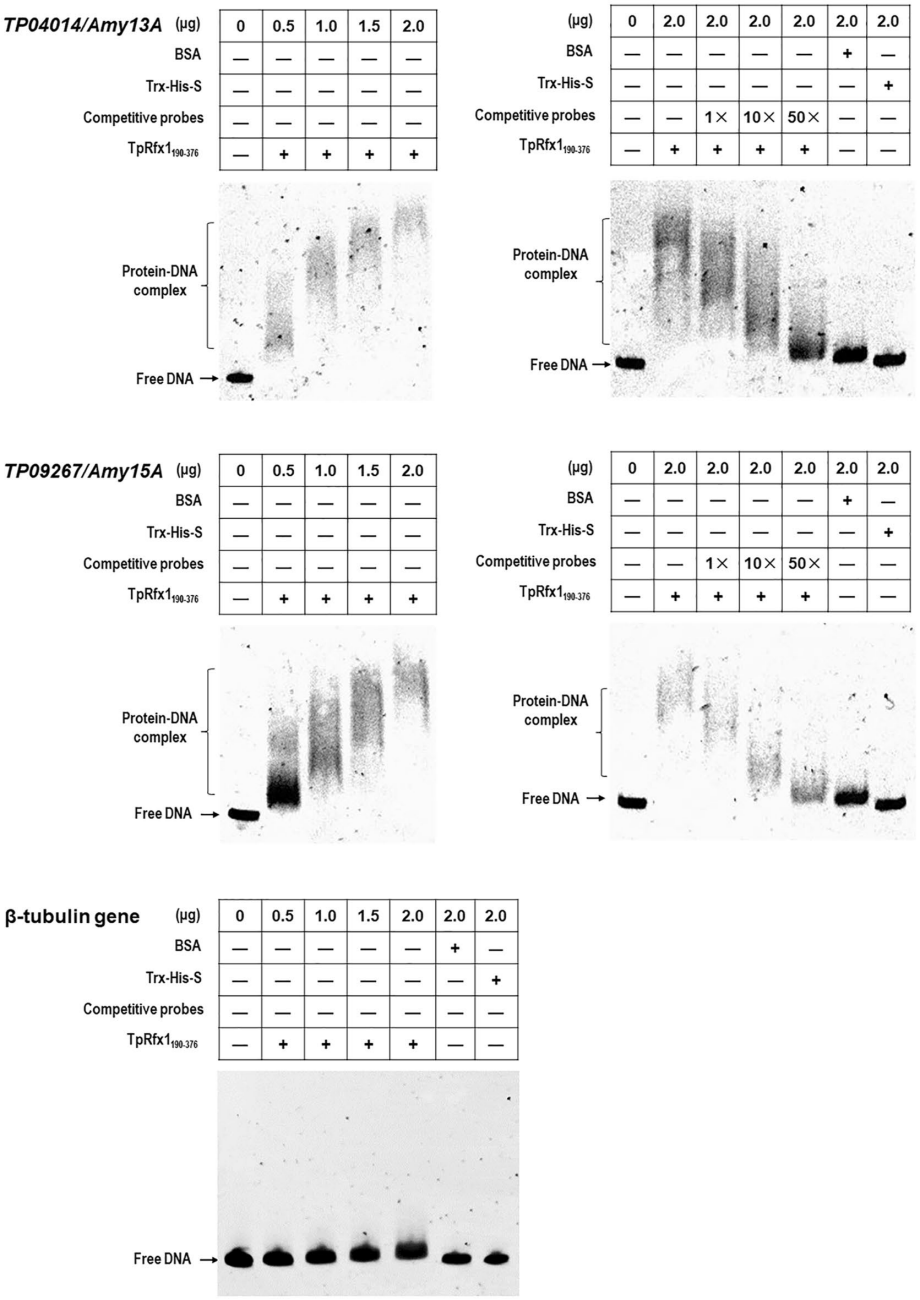
EMSA revealing interactions between TpRfx1 DNA-binding domain and the promoter regions of amylase genes. The indicated amounts of purified TpRfx1_190-367_ were mixed with about 150 ng of FAM-labeled EMSA probes. EMSA probes lacking the FAM label and the promoter region of β-tubulin gene were used for competitive EMSA and as a negative control, respectively. BSA alone and TRX-His-S from *E. coli* cells containing the empty vector pET-32a(+) were used as controls

**Fig. 7:**
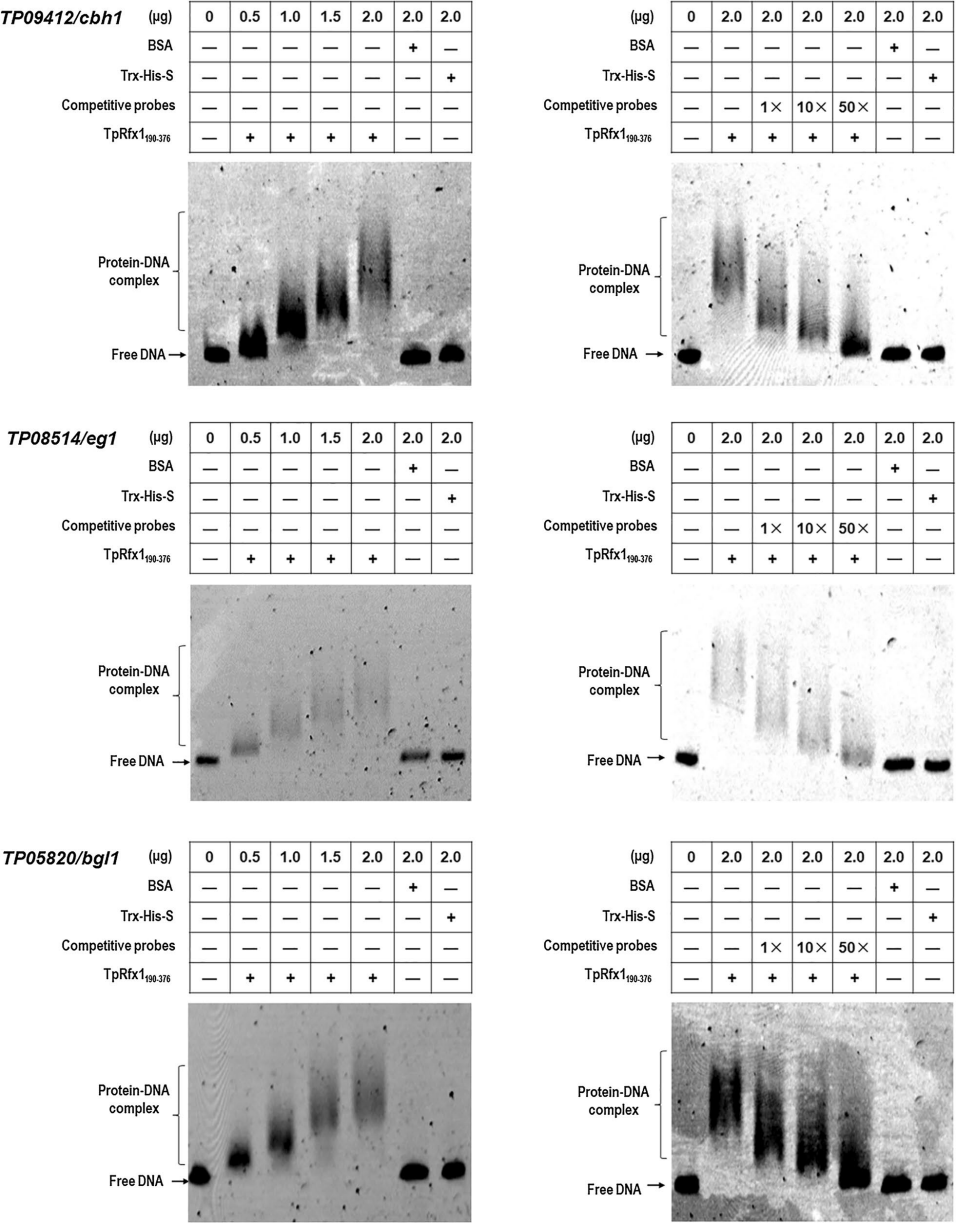
TpRfx1 binding to the promoter regions of cellulase genes. The indicated amounts of purified TpRfx1_190-367_ were mixed with about 150 ng of FAM-labeled EMSA probes. EMSA probes lacking the FAM label and the promoter region of β-tubulin gene were used for competitive EMSA and as a negative control, respectively. BSA alone and TRX-His-S from *E. coli* cells containing the empty vector pET-32a(+) were used as controls

To confirm whether TpRfx1 directly or indirectly regulates targeted gene expression, in vitro binding experiments were performed using an electrophoretic mobility shift assay (EMSA). The predicted DNA-binding domain of TpRfx1 (TpRfx1_190-376_) was expressed in *Escherichia coli*, and the recombinant protein TRX-His-S-tagged TpRfx1_190-376_ was subsequently purified using a nickel-affinity column. The 6-FAM-tagged DNA fragments (~ 1000 bp) located in the promoter regions of the five selected target genes, including two amylase genes (*TP04014* and *TP09267*), three cellulase genes (*TP09412*, *TP08514*, and *TP05820*), were PCR amplified using specific primer pairs (Additional file 3: Table S2). EMSA experiments revealed shifted bands representing TpRfx1_190-376_-DNA complexes in all mixtures of TpRfx1_190-376_ and the promoter regions of the tested genes. Moreover, we observed increase in band size along with increases in TpRfx1_190-376_ mass used in the gels (0.5–2.0 µg). Subsequently, we performed competitive EMSA using the same DNA fragments lacking the FAM tag, with results revealing weaker binding affinities and smaller band sizes associated with the shifted bands. As expected, no shifted bands were observed in mixtures comprising TpRfx1_190-376_ and the promoter region for *β*-*tubulin* gene or bovine serum albumin (BSA), the purified TRX-His-S fusion protein, and any tested target genes (Figs. 6, 7). These results suggested that TpRfx1_190-376_ specifically bound all five tested DNA sequences from the promoter regions of the amylase and cellulase genes in vitro.

### The DNA-binding sequence targeted by TpRfx1

**Fig. 8:**
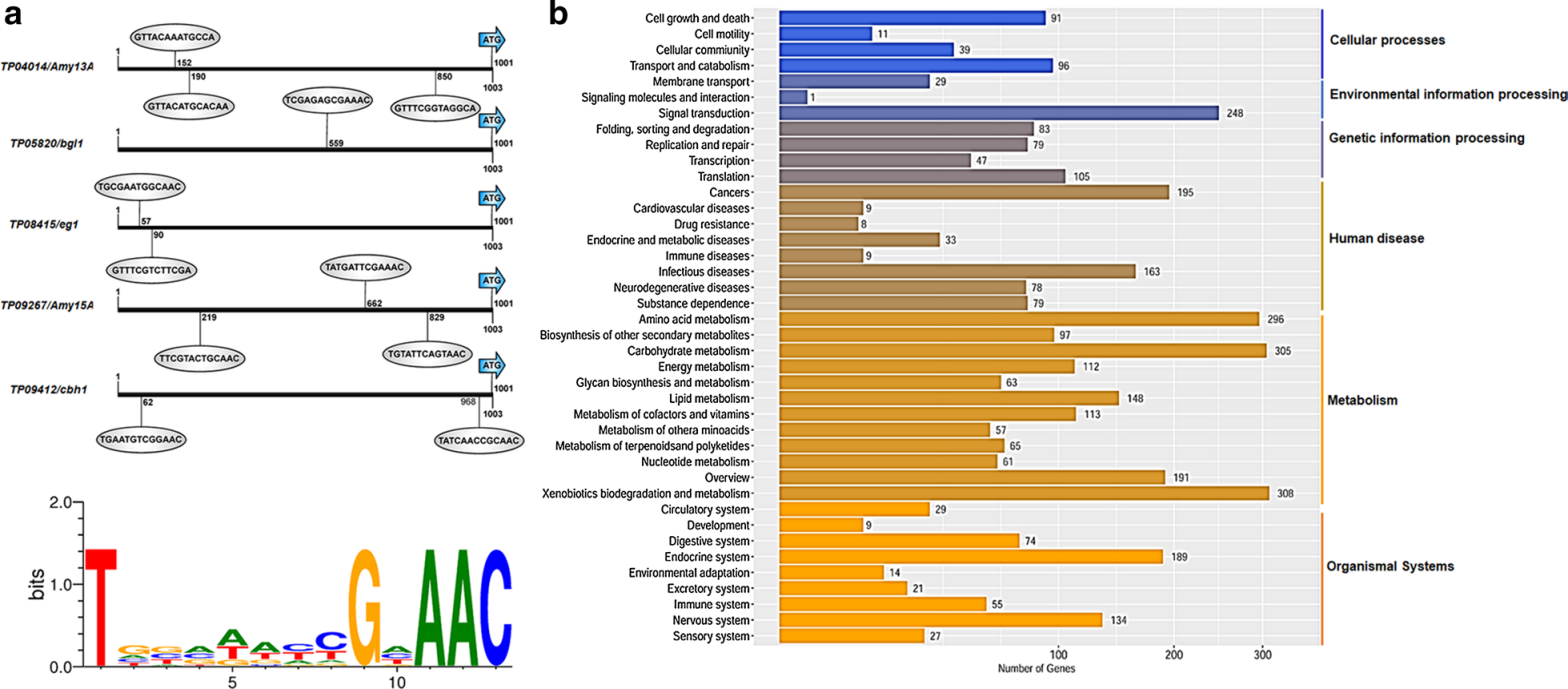
Identification of the TpRfx1-binding motif and putative TpRxf1 target genes in the genome of *T. pinophilus* 1-95. **a** The TpRfx1-binding sequence in experimentally confirmed TpRfx1 targets, including amylase genes (*TP04014*/*Amy13A* and *TP09267*/*Amy15A*) and cellulase genes (*TP09412*/*cbh1*, *TP08514*/*eg1*, and *TP05820*/*bgl1*). **b** Functional analysis of putative TpRfx1 target genes according to Kyoto Encyclopedia of Genes and Genomes (KEGG) annotations

TpRfx1 regulates amylase and cellulase gene expression by directly binding to their promoter regions. To determine the targeted DNA sequence in the promoter regions, a consensus RFX-binding sequence (RTHNYYN_0-3_RGNAAC) identified previously [2, 18] was used to search 1-kb regions of the 5′ untranslated regions of the tested amylase genes (*TP04014* and *TP09267*) and cellulase genes (*TP05820*, *TP08514*, and *TP09412*). The results identified at least one similar binding sequence (TN_3_DN_3_GNAAC) in each target gene, with the “T” nucleotide and the “GNAAC” sequence highly conserved (Fig. 8a).

This putative DNA-binding sequence targeted by TpRfx1 was used to search the genome of *T*. *pinophilus* 1-95 [3] using the MEME suite 4.12.0 (http://meme-suite.org/index.html), with results indicating that 1-kb regions of the promoters for 2169 genes contained at least one instance of this putative TpRfx1-binding sequence. Pathway classification revealed that these target genes are mainly involved in metabolism (xenobiotics biodegradation and metabolism, carbohydrate metabolism, and amino acid metabolism) and signal transduction (Fig. 8b).

## Discussion

In this study, we screened and identified novel TFs regulating amylase and cellulase gene expression in *T. pinophilus* through comparative transcriptome and molecular genetic analyses. Comparative transcriptomics combined with genetic analyses are considered efficient methods for screening novel TFs, as described in the identification of the TFs RCA-1, PoxCxrA, PoxCxrB, and PoxNsdD in *Neurospora crassa* and *P*. *oxalicum* [19, 20]. In the present study, seven novel TFs were identified as regulating amylase production in *T*. *pinophilus* under SCS induction, with most of these containing a Zn2Cys6 domain. Furthermore, we identified TpRfx1 belonging to the RFX TF family as an essential positive regulator for amylase gene expression. RFX1 proteins are evolutionarily conservative according to their RFX DNA-binding domain and exist broadly in eukaryotes from yeast to humans. The RFX1 protein regulates the immune response in mammals and is response to infection by human hepatitis B virus, as well as cell-differentiation events in fission yeast [10]. Recently, RFX1 proteins were identified as being involved in controlling the cell growth and morphogenesis of the filamentous fungi *T*. *marneffei* [12], as well as the biosynthesis of secondary catabolites, such as β-lactam, cephalosporin C, and penicillin, in *P. chrysogenum* and *A. chrysogenum* [13, 21]. In the present study, we expanded the regulatory roles of RFX1 proteins in amylase and cellulase production in the filamentous fungus *T. pinophilus* for the first time, suggesting diversity in the functions of conserved RFX1 proteins as regulators of gene expression.

TpRfx1 regulates amylase and cellulase gene expression by directly binding to their promoter regions, which harbor at least one putative DNA-binding site specific for this TF. The putative TpRfx1 DNA-binding sequence (TN_3_DN_3_GNAAC) shares the nucleotide “T” and the five nucleotides “GNAAC” with the consensus RFX-binding sequence (RTHNYYN_0-3_RGNAAC) identified previously [12, 18], implying that these nucleotides are required for interactions between RFX1 TFs and their target genes.

In addition to the regulatory roles of RFX proteins RFX1 and CPCR1 reported in *T*. *marneffei* and *A. chrysogenum* [12, 21], TpRfx1 positively regulated hyphae growth and conidiation of *T. pinophilus*. Moreover, *TpRfx1* deletion led to reduced transcription of important amylase and cellulase genes, resulting in the decreased production of extracellular amylases or cellulases to degrade SCS or cellulose, thereby leading to impaired cell differentiation and conidiation.

**Fig. 9:**
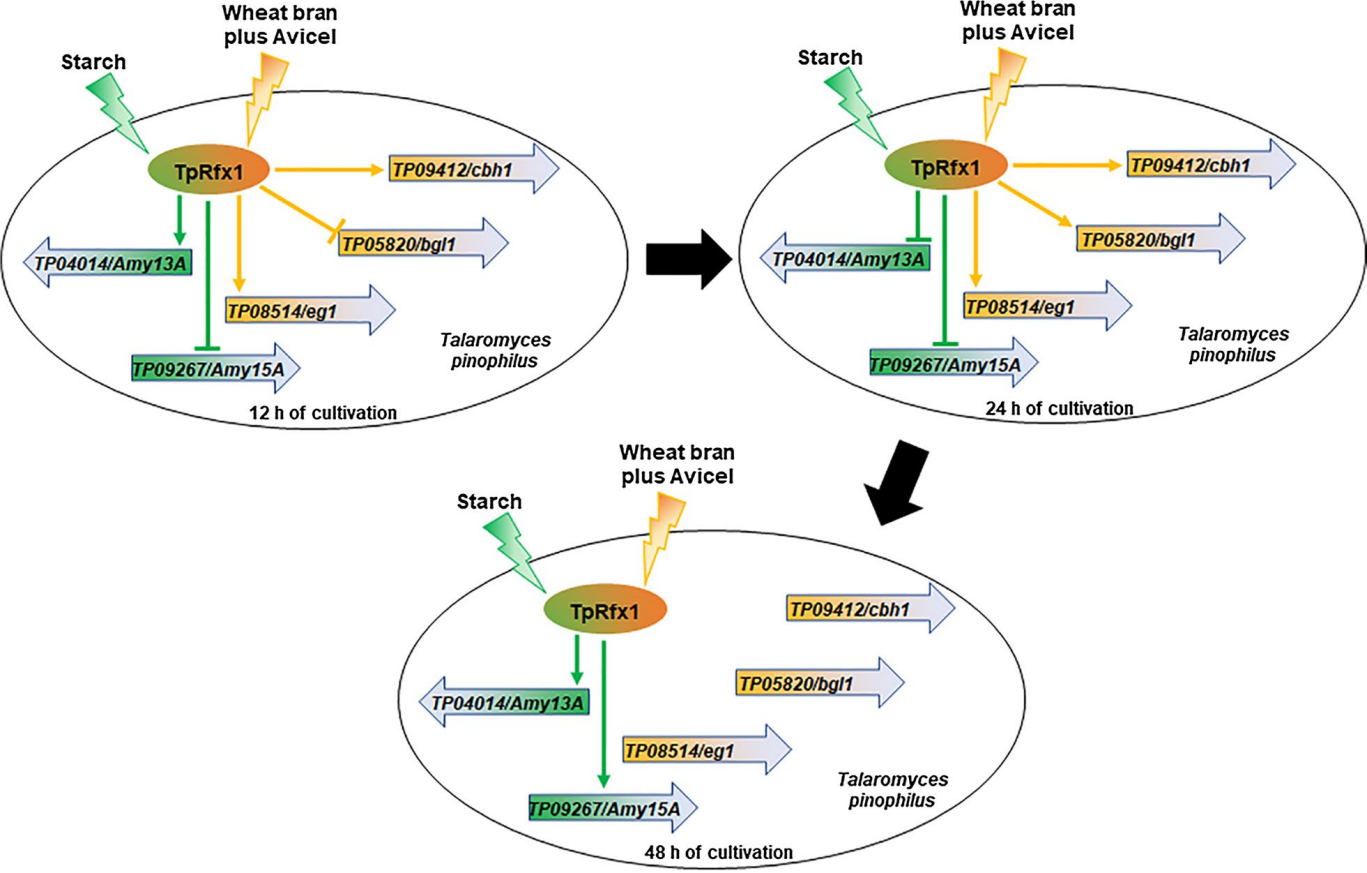
TpRfx1-mediated regulation of amylase and cellulase gene expression in *T. pinophilus* in the presence of SCS and WA

*Talaromyces pinophilus* strain 1-95 secretes extracellular enzymes in a carbon source-dependent manner. For example, high-yield amylase production, but not cellulase production, is observed in the presence of SCS medium (data not shown). Here, we found that TpRfx1 regulated the expression of different genes encoding plant biomass-degrading enzymes, such as amylases and cellulases, dependent on the type of carbon source used for induction. Our findings suggested the existence of a novel TpRfx1-mediated real-time network involved in regulating amylase and cellulase gene expression in *T. pinophilus* in the presence of SCS and WA, respectively (Figs. 5, 9). In the presence of SCS, TpRfx1 initiated the expression of α-amylase (*TP04014*/*Amy13A*) and inhibited glucoamylase (*TP09267*/*Amy15A*) expression during the early induction stage. Subsequently, transcription of both amylase genes was repressed by TpRfx1. During the later stage, TpRfx1 functioned as an activator to promote the expression of both genes. Interestingly, the transcription of important cellulase genes was not regulated by TpRfx1 in the presence of SCS. By contrast, in the presence of WA, the expression of both CBH (*TP09412*/*cbh1*) and EG (*TP08514*/*eg1*) was enhanced by TpRfx1, whereas BGL (*TP05820*/*bgl1*) expression was repressed at 12-h post-induction. Subsequently, at 24-h post-induction, TpRfx1 enhanced the transcription of all the three cellulase genes; however, at later stages (i.e., 48-h post-induction), TpRfx1 did not regulate the expression of these three cellulase genes.

In *S*. *pombe*, the TpRfx1 homologous protein Sak1 controls cAMP-dependent protein kinase-mediated exit from the mitotic cell cycle [15]. In *P. oxalicum*, the G protein-cAMP pathway positively regulates the expression of amylase genes, such as *amy15A*, in the presence of either starch or cellulose while negatively regulating *cel7A*-*2* expression during early induction by controlling the expression of the *AmyR* [22]. Recently, Zhang et al. [17] reported that *TpAmyR* (*TP09286*) positively regulates important amylase genes in *T. pinophilus*, which agrees with the regulatory roles reported for AmyR in other filamentous fungi, such as *P*. *oxalicum* [6] and *Aspergillus* sp. [23].

Additionally, another TpRfx1 homologous protein, Crt1, can bind regions of DNA-damage-response genes, including *RNR2*-*4* and *HUG1*, to recruit the chromatin-repression complex SSN6-TUP1 and histone deacetylases to maintain these genes in a repressed state during methyl methanesulfonate induction of *Saccharomyces cerevisiae* [24, 25]. Conversely, Crt1 also facilitates the recruitment of the chromatin-remodeling complexes SWI/SNF and TFIID to promote chromatin remodeling and full assembly of the preinitiation complex to activate ribonucleotide reductase gene expression [26–28]. Spt-Ada-Gcn5 acetyltransferase complex subunit-encoding genes, such as *Gcn5* and *Ada2*, activates the transcription of cellulase genes in *T. reesei* and regulates mycelial growth [29, 30]. Additionally, the SWI/SNF complex subunit-encoding gene *HepA* (*Swi6* in *S. cerevisiae*) is a positive regulator required for cellulase and amylase gene expression in *P*. *oxalicum* [31], and *Snf2* expression is significantly upregulated in *T*. *reesei* strain ∆*Cre1* and possibly involved in cellulase gene repression by Cre1 [32]. However, these regulatory mechanisms in *T. pinophilus* remained unclear. To augment our findings, additional investigation is necessary to elucidate the regulatory mechanisms associated with TpRfx1 regarding its mediation of cellulase and amylase gene expression in *T. pinophilus* under starvation conditions.

## Conclusions

Altogether, in this study, we expanded the novel regulatory roles of the RFX proteins in the regulation of genes encoding plant biomass-degrading enzymes including amylase and cellulase in *T. pinophilus* for the first time, which provides a novel insight into deeply understanding of molecular regulatory mechanism of the expression of functional enzyme genes at transcriptional level in fungi and a potential target for genetic engineering processes.

## Methods

### Microbial strains and culture conditions

Fungal strains used in this study included the parental strain ∆*TpKu70*, the deletion mutants for the 30 candidate genes (Additional file 1: Table S1), and the complementary strain *CTpRfx1*. Fungal strains were maintained on PDA plates at 4 °C. For sporulation and mycelial growth (for DNA extraction), PDA medium and liquid complete medium [(g/L) d-glucose 10, peptone 2, yeast extract 1, acid-hydrolyzed casein 1, and 50 mL 20× nitrate-containing NaNO_3_ 120, KCl 10.4, MgSO_4_·7H_2_O 10.4, KH_2_PO_4_ 30.4 (pH 6.5)] were inoculated and subsequently incubated at 28 °C for 6 days [33]. For enzymatic activity measurement and RT-qPCR analyses, approximately 1 × 10^8^ fresh spores were pre-grown in 100 mL glucose medium at 28 °C for 24 h. Certain amounts of mycelia were harvested, transferred into standard liquid medium (SLM) [(g/L) tryptone 5, KH_2_PO_4_ 3, (NH_4_)_2_SO_4_ 2.5, MgSO_4_·7H_2_O 0.2, CaCl_2_ 0.13, and FeSO_4_ 0.0255] containing 1% (w/v) SCS (Sigma-Aldrich, St. Louis, MO, USA) or 1% (w/v) wheat bran plus 1% (w/v) Avicel (Sigma-Aldrich) and incubated at 28 °C for 2–4 days. For mycelial-biomass determination, SLM containing 1% (w/v) d-glucose was used and cultivated at 28 °C for 84 h. For phenotypic analyses, solid SLM containing 1% (w/v) SCS was cultivated at 28 °C for 8 days, with PDA used as a control. *E. coli* Trans1-T1 and DE3 were used as cloning and expression hosts, respectively, and cultured at 37 °C in Luria–Bertani medium.

### Total DNA and RNA extraction

Total fungal DNA and RNA were extracted from mycelia harvested from PDA plates or liquid media, as previously described [33]. Briefly, harvested mycelia were ground into flour in liquid nitrogen and lysed using lysate reagent [20 mM sodium acetate trihydrate, 40 mM Tris–HCl, 10 mM ethylenediaminetetraacetic acid, and 1% sodium dodecyl sulfate (pH 8.0)]. Total DNA was separated from the lysed suspension after removing protein with phenol–chloroform and isopropanol precipitation. Additionally, a TRIzol RNA kit (Life Technologies, Carlsbad, CA, USA) was used to extract total RNA according to manufacturer instructions.

### Construction of deletion cassettes and *T. pinophilus* transformation

Deletion cassettes were constructed using double-joint PCR, as described previously [17]. Each deletion cassette contained ~ 2 kb of the 5′- and 3′-flanking regions of the target gene and 2.8 kb of the benomyl-resistance gene (*benA*) and was amplified using corresponding primer pairs (Additional file 3: Table S2). The amplified product was introduced into ∆*TpKu70* protoplasts [33] and transformants were confirmed by PCR or Southern hybridization analysis using specific primers (Additional file 3: Table S2).

### Mutant complementation

Complementation of ∆*TpRfx1* was performed according to previously described methods [20]. Briefly, a gene-complementary cassette was constructed through fusion PCR and comprised the upstream and downstream DNA sequences of *TpRfx1*, the predicted termination sequence of *TP06064*, and the bleomycin-resistance gene (*ble*), all of which were PCR amplified using corresponding primer pairs (Additional file 3: Table S2 and Additional file 4: Fig. S2). The resulting cassette was introduced into ∆*TpRfx1* protoplasts, and complementary strains were confirmed with PCR using specific primer pairs (Additional file 3: Table S2).

### Southern hybridization

The ∆*TpRfx1* strain was confirmed with Southern hybridization analysis according to previously described methods [33]. The genomic DNA of the ∆*TpRfx1* strain was extracted and digested with *Sac*I (TaKaRa Bio Inc., Dalian, China), and the generated DNA fragments were separated on 0.8% agarose gels, followed by transfer to a Hybond-N^+^ nylon membrane (GE Healthcare Ltd., Little Chalfont, UK). DIG-high detection starter kit (Life Technologies) was used to investigate the hybridized bands using a DIG-high-labeled DNA probe amplified using the primers indicated in Additional file 3: Table S2.

### Enzyme activity assay and determination of intracellular protein concentration

Amylase and cellulase (FPase, CMCase, xylanase, pNPCase, and pNPGase) activities were determined as previously described [17, 33]. Briefly, suitably diluted crude enzyme solution was added to 0.1 M citrate–phosphate buffer (pH 5.0) containing 1% of different substrates, including SCS, filter paper, carboxymethyl cellulose, and xylan, and incubated at 55 °C for 30 min and 50 °C for 60, 30 and 10 min, respectively. The generated reducing sugars were determined using 3,5-dinitrosalicylic acid method [34] at 540 nm. One unit of enzymatic activity (U) was defined as the amount of enzyme required to produce 1 µmol of reducing sugar per min from the reaction substrates.

In addition, *p*-nitrophenyl-β-d-cellobioside (pNPC) and *p*-nitrophenol-α-d-glucopyranoside (pNPG) were used as the substrates for measurement of pNPCase and pNPGase activities, respectively. A 140 µL mixture containing 14 µL of 25 mM pNPC or pNPG solution, 116 µL of citrate buffer (100 mM, pH 5.0), and 10 µL of diluted crude cellulase was incubated at 50 °C for 15 min, and then 70 µL of sodium carbonate (0.4 M) was added into the mixture to stop the reaction. The produced *p*-nitrophenol was measured at 410 nm, and 1 U of enzymatic activity (U) was defined as the amount of enzyme that produced 1 µmol of *p*-nitrophenol per min from the appropriate substrate. Triplicate independent experiments were performed for each sample.

Determination of intracellular protein concentration in fungal mycelia was conducted according to previously described methods [35]. The collected mycelia were milled in liquid nitrogen and added to protein extract buffer. Protein concentration was measured based on the Bradford method.

### Determination of dry biomass weight

Fresh spore suspensions at concentrations of 1.0 × 10^8^ spores/mL were inoculated into 100 mL of glucose or starch liquid medium, respectively, and cultivated at 28 °C for 24 h to 84 h. The hypha was collected by vacuum suction filter and then dried at 50 °C to a constant weight.

### RT-qPCR analysis

The PrimeScript RT reagent kit (TaKaRa) was used for RT-qPCR analysis according to manufacturer instructions. Each PCR was performed in a final volume of 20 µL, including 10 μL of SYBR Premix ExTaq II, 0.8 μL of 10 μM forward primer, 0.8 μL of 10 μM reverse primer, 2.0 μL of cDNA, and 6.4 μL of sterile water. Reaction procedures were as follows: initial denaturation for 2 min at 95 °C, followed by 40 cycles of 10 s at 95 °C and 30 s at 61 °C. The fluorescence signal was measured at the end of each extension step at 80 °C. Relative expression levels of the tested genes were calculated according to previously described methods [17].

### Expression of the DNA sequence encoding the putative DNA-binding domain of TpRfx1 and purification of the translated product

The DNA sequence encoding the putative DNA-binding domain TpRfx1_190-376_ was amplified with PCR using the primer pairs TP06128-domain-F and TP06128-domain-R (Additional file 3: Table S2). The DNA fragment was digested by *Not*I and *Hin*dIII and cloned into vector pET32a(+) digested with the same restriction endonucleases. The generated pET32a-TpRfx1_190-376_ was introduced into *E. coli* Trans-DE3 cells and after isopropyl-β-d-thiogalactopyranoside induction, the recombinant proteins fused with TRX, His, and S tags were purified using TALON metal-affinity resin (Clontech, Palo Alto, CA, USA). The strain containing the empty vector pET32a(+) was cultivated as a control.

### In vitro EMSA

EMSA was performed as previously described [20]. Briefly, approximately 1-kb DNA fragments from the promoter regions of the tested genes were used as probes following amplification by PCR using specific primers (Additional file 3: Table S2). FAM-tagged EMSA probes were mixed with various amounts (0–2.0 μg) of TpRfx1_190-367_ in binding buffer [0.1 mg/mL BSA, 20 mM Tris–HCl (pH 8.0), 5% glycerol, 50 mM KCl, 1 mM DTT, and 2 μg sheared salmon sperm DNA] and incubated for 20 min. Polyacrylamide Tris–acetic acid–EDTA gel electrophoresis and the Bio-Rad ChemiDoc MP imaging system (Bio-Rad Laboratories, Hercules, CA, USA) were used for the separation and detection of protein–DNA complexes, respectively. EMSA probes without FAM labels were used for competitive EMSA. BSA alone and TRX-His-S from *E. coli* cells transformed with the empty vector pET-32a(+) were used as controls.

### Phylogenetic analysis

TpRfx1 and its homologous proteins were phylogenetically analyzed using MEGA 7.0 software [36]. A phylogenetic tree was constructed based on the neighbor-joining method and a Poisson correction model. In this process, the bootstrap values were calculated when 1000 replicates were established.

### Statistical analysis

All experimental data involving enzyme production and gene transcription were statistically analyzed using Microsoft Excel (Office 2016; Microsoft, Redmond, WA, USA). Significance analyses among samples were performed by Student *t* test, and a *P *< 0.05 was considered significant.

## Additional files

**Additional file 1: Table S1.** List of candidate regulatory genes for regulating amylase production of *T. pinophilus*.

**Additional file 2: Figure S1.** Confirmation analysis of the deletion mutants of 23 candidate genes in *T. pinophilus* mutant ∆*TpKu70* as the parental strain. A–W. PCR confirmation analysis of (A) ∆*TP00297*, (B) ∆*TP02310*, (C) ∆*TP02980*, (D) ∆*TP03450*, (E) ∆*TP03988*, (F) ∆*TP05236*, (G) ∆*TP05746*, (H) ∆*TP05940*, (I) ∆*TP06128*, (J) ∆*TP06213*, (K) ∆*TP06945*, (L) ∆*TP06973*, (M) ∆*TP07409*, (N) ∆*TP08445*, (O) ∆*TP08615*, (P) ∆*TP08885*, (Q) ∆*TP09107*, (R) ∆*TP09505*, (S) ∆*TP09510*, (T) ∆*TP09544*, (U) ∆*TP09568*, (V) ∆*TP095904*, (W) ∆*TP12095*. Line M: 1-kb DNA marker, Lanes 1–3: three transformants constructed for each candidate gene, Lane 4: ∆*PoxKu70*, Lane 5: ddH_2_O. The PCR products for each deletion mutant included the production of each target gene (Top), the production of the fragment on the left of the target gene (Middle) and the production of the fragment on the right of the target gene. X. Southern hybridization confirmation of the mutant ∆*TP06128*. The probe was amplified from the 5′-flanking sequence of *TP06128*. Lane M: 1-kb DNA marker, Lane 1: genomic DNA from ∆*PoxKu70* as the control, Lanes 2–4, genomic DNA from three transformants of ∆*TP06128*.

**Additional file 3: Table S2.** The primers used in this study.

**Additional file 4: Figure S2.** Complementation of the mutant ∆*TP06128*. A. *TP06128* complementary DNA cassette integrated into genome of ∆*TP06128* through homologous recombination. B–D. PCR confirmation of the complementary strain. PCR products were amplified with specific primer pairs TP06128-V-F/TP06128-V-R (B), CTP06128-L-F/TP06064ter-R (C) and TP06064ter-F/CTP06128-R-R (D), respectively. Lane M: 1-kb DNA marker, Lanes 1–3: genomic DNA from three transformants of complementary strain, Lane +: genomic DNA from the ∆*TpKu70* as the positive control, and Lane −: genomic DNA from the ∆*TP06128* as the negative control.

## Abbreviations

PDA: potato dextrose agar
SLM: standard liquid medium
EG: endo-β-1:4-glucanase
CBH: cellobiohydrolase
BGL: β-glucosidase
TF: transcription factor
RFX: regulatory factor X
FPase: filter paper cellulase
CMCase: carboxymethylcellulase
pNPCase: *p*-nitrophenyl-β-cellobiosidase
pNPGase: *p*-nitrophenyl-β-glucopyranosidase
RT-qPCR: real-time quantitative reverse transcription PCR
SCS: soluble corn starch
EMSA: electrophoretic mobility shift assay
FAM: 6-carboxyfluorescein
BSA: bovine serum albumin
