# Supplementary material for: The transcription factor TpRfx1 is an essential regulator of amylase and cellulase gene expression in Talaromyces pinophilus
Source: Biotechnol Biofuels. 2018 Oct 8;11:276. doi: 10.1186/s13068-018-1276-8 (PMC6174557; doi:10.1186/s13068-018-1276-8)
Supplement: Supplementary file 1 — Additional file 1: Table S1. List of candidate regulatory genes for regulating amylase production of T. pinophilus. [file 13068_2018_1276_MOESM1_ESM.pdf]

Additional file 1: Table S1. List of candidate regulatory genes for regulating amylase production of *T. pinophilus* 1-95

Gene ID	Domain description of predicted protein
TP00297	Zn2Cys6 type; Fungal_Trans
TP00989	Zinc finger, PARP-type
TP01028	Zinc finger; C2H2-type
TP02294	Basic-leucine zipper (bZIP) transcription factor
TP02310	Zn2Cys6 type; Fungal_Trans
TP02980	Centromere protein B; DNA-binding region
TP03450	DNA-binding; APSES Fungal_Trans
TP03988	Zn2Cys6 type; Fungal_Trans
TP04628	Zn2Cys6 type; Fungal_Trans
TP04707	Zinc finger; Zn2Cys6 type
TP05119	Zinc finger, Zn2Cys6 type; Fungal_Trans
TP05236	Zn2Cys6 type; Fungal_Trans
TP05290	Zinc finger, Zn2Cys6 type; Fungal_Trans
TP05746	Zn2Cys6 type; Fungal_Trans
TP05940	Winged helix repressor DNA-binding
TP06038	Zinc finger, Zn2Cys6 type; Fungal_Trans
TP06128	RFX DNA-binding domain
TP06213	Zn2Cys6 type; Fungal_Trans
TP06945	Zn2Cys6 type; Fungal_Trans
TP06973	Zn2Cys6 type; Fungal_Trans
TP07409	Zn2Cys6 type; Fungal_Trans
TP08445	Zn2Cys6 type; Fungal_Trans
TP08615	Zn2Cys6 type; Fungal_Trans
TP08885	Zinc finger; C2H2-type
TP09107	Zn2Cys6 type; Fungal_Trans
TP09286	Zinc finger, Zn2Cys6 type; Fungal_Trans (AmyR)
TP09505	Zn2Cys6 type; Fungal_Trans
TP09510	Zn2Cys6 type; Fungal_Trans
TP09544	Zn2Cys6 type; Fungal_Trans
TP09568	Zinc finger; C2H2-type
TP09590	Zn2Cys6 type; Fungal_Trans
TP12095	Zn2Cys6 type; Fungal_Trans
TP13060	Zinc finger, C2H2-type; Fungal_Trans
