# Supplementary figures and images for: The transcription factor TpRfx1 is an essential regulator of amylase and cellulase gene expression in Talaromyces pinophilus
Source: Biotechnol Biofuels. 2018 Oct 8;11:276. doi: 10.1186/s13068-018-1276-8 (PMC6174557; doi:10.1186/s13068-018-1276-8)

**A**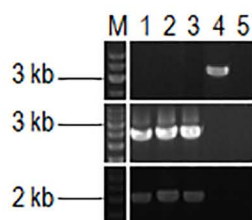**B**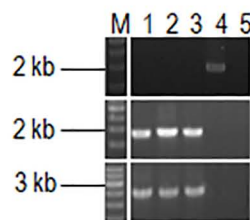**C**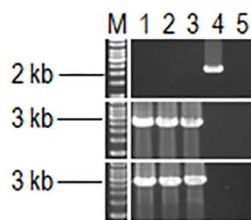**D**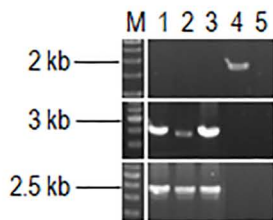**E**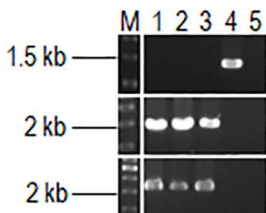**F**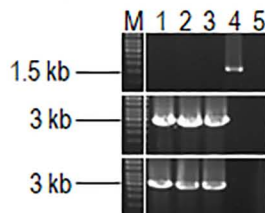**G**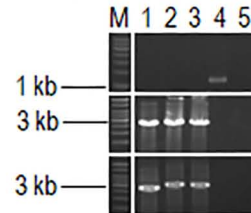**H**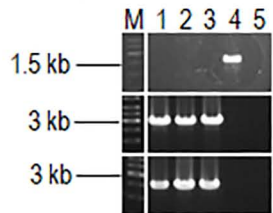**I**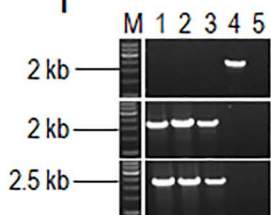**J**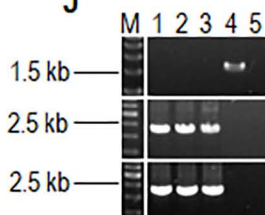**K**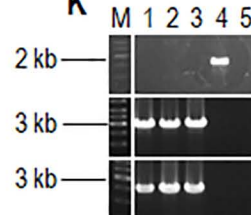**L**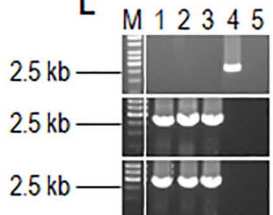**M**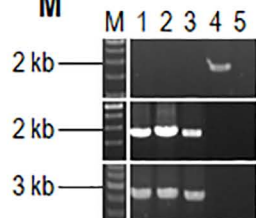**N**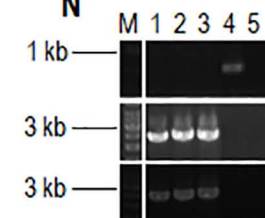**O**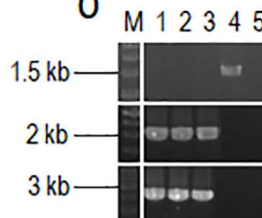**P**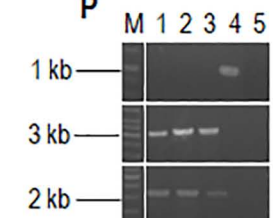**Q**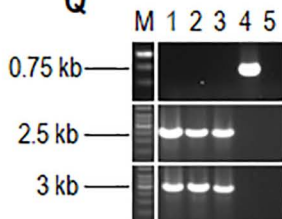**R**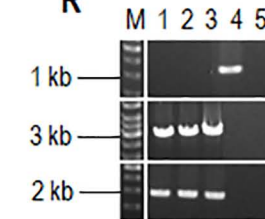**S**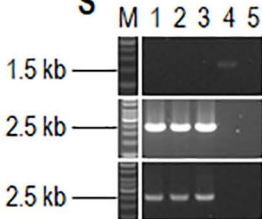**T**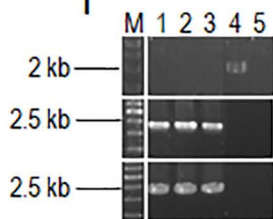**U**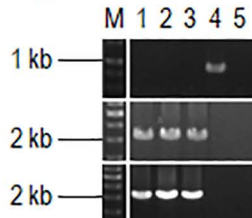**V**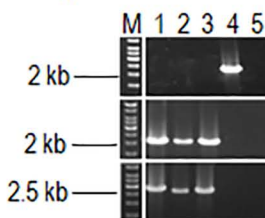**W**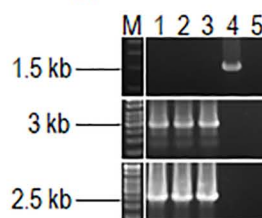**X**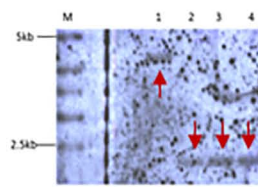

Supplement: Supplementary file 2 — Additional file 2: Figure S1. Confirmation analysis of the deletion mutants of 23 candidate genes in T. pinophilus mutant ∆TpKu70 as the parental strain. A–W. PCR confirmation analysis of (A) ∆TP00297, (B) ∆TP02310, (C) ∆TP02980, (D) ∆TP03450, (E) ∆TP03988, (F) ∆TP05236, (G) ∆TP05746, (H) ∆TP05940, (I) ∆TP06128, (J) ∆TP06213, (K) ∆TP06945, (L) ∆TP06973, (M) ∆TP07409, (N) ∆TP08445, (O) ∆TP08615, (P) ∆TP08885, (Q) ∆TP09107, (R) ∆TP09505, (S) ∆TP09510, (T) ∆TP09544, (U) ∆TP09568, (V) ∆TP095904, (W) ∆TP12095. Line M: 1-kb DNA marker, Lanes 1–3: three transformants constructed for each candidate gene, Lane 4: ∆PoxKu70, Lane 5: ddH2O. The PCR products for each deletion mutant included the production of each target gene (Top), the production of the fragment on the left of the target gene (Middle) and the production of the fragment on the right of the target gene. X. Southern hybridization confirmation of the mutant ∆TP06128. The probe was amplified from the 5′-flanking sequence of TP06128. Lane M: 1-kb DNA marker, Lane 1: genomic DNA from ∆PoxKu70 as the control, Lanes 2–4, genomic DNA from three transformants of ∆TP06128. [file 13068_2018_1276_MOESM2_ESM.pdf]

**A**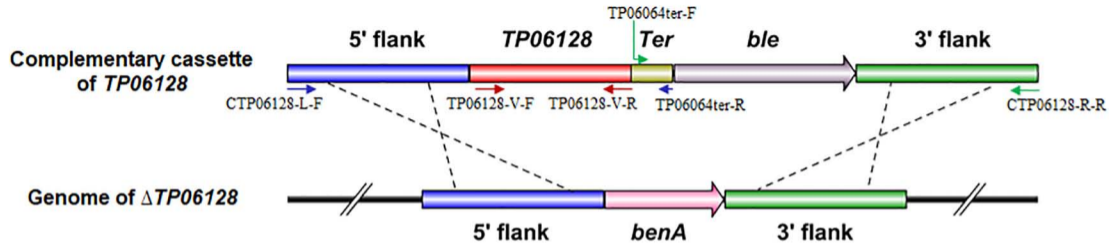**B**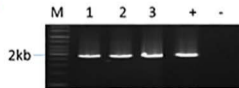**C**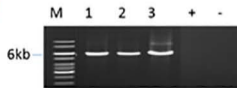**D**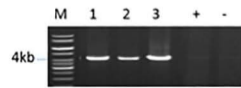

Supplement: Supplementary file 4 — Additional file 4: Figure S2. Complementation of the mutant ∆TP06128. A. TP06128 complementary DNA cassette integrated into genome of ∆TP06128 through homologous recombination. B–D. PCR confirmation of the complementary strain. PCR products were amplified with specific primer pairs TP06128-V-F/TP06128-V-R (B), CTP06128-L-F/TP06064ter-R (C) and TP06064ter-F/CTP06128-R-R (D), respectively. Lane M: 1-kb DNA marker, Lanes 1–3: genomic DNA from three transformants of complementary strain, Lane +: genomic DNA from the ∆TpKu70 as the positive control, and Lane −: genomic DNA from the ∆TP06128 as the negative control. [file 13068_2018_1276_MOESM4_ESM.pdf]
