# Supplementary material for: The transcription factor TpRfx1 is an essential regulator of amylase and cellulase gene expression in Talaromyces pinophilus
Source: Biotechnol Biofuels. 2018 Oct 8;11:276. doi: 10.1186/s13068-018-1276-8 (PMC6174557; doi:10.1186/s13068-018-1276-8)
Supplement: Supplementary file 3 — Additional file 3: Table S2. The primers used in this study. [file 13068_2018_1276_MOESM3_ESM.pdf]

Additional file 3: Table S2. The primers used in this study.

Primer name	Sequence (5' →3')
**Primers used for RT-qPCR**	
RT-TP11464-F	AAGGGTTGCGACGGTTTC
RT-TP11464-R	AGGGAGTATCGGGGTTTGTG
RT-TP12265-F	CAAATGCGGTGAGTAGTGCTG
RT-TP12265-R	TCAAACATTCACCTCCCAGC
RT-TP03913-F	GAATCGGCAAAGGCACACG
RT-TP03913-R	GCATTAGGCACAAGACTGACACC
RT-TP05120-F	AGTTCTGGTTACGCAAGGGC
RT-TP05120-R	ATCTGGGAAGTCTGACGGCT
RT-TP00071-F	CCATACTGGGGTTTCGGTTT
RT-TP00071-R	CGTTGAAGGCAGCATAATCC
RT-TP05786-F	TGTTGCTTGTTTGATTTCCCC
RT-TP05786-R	CATCGGCATAGGCTCGGTT
RT-TP000293-F	TGCCACAAGCACGGAATG
RT-TP000293-R	CGCCAGAAATACCAATCGC
RT-TP000938-F	CATCTCAACGCAACGCTACTG
RT-TP000938-R	GAGCCACTTTGAGGATGTTAGC
RT-TP01354-F	ACCATTCGTCTTGACTCGCA
RT-TP01354-R	CCACCAATGTCGTGTCCGT
RT-TP03337-F	CCAACGGCAAATACACACCC
RT-TP03337-R	CGCTGTCGTGATTGTCAAGGTA
RT-TP04937-F	TCTCAGCCACTGCTTCCACG
RT-TP04937-R	CCCAGTAACCCCTGATTCGG
RT-TP04013-F	AATGTTCAGACAGGGCACGA
RT-TP04013-R	GACCGATAATAAATGGACGCTT
RT-TP03368-F	GGAATGCGTGGGTCAAGTCA
RT-TP03368-R	CGCCATAATCCAGTTCTCCAATAC
RT-TP03580-F	TTGGGATGCGGTGCTGAA
RT-TP03580-R	TCTTCTGGTTTCGTGAGGTCG
RT-TP07411-F	TGATGGTTGATGTGGTGGCG
RT-TP07411-R	GACTGGTTGAACGGAACAAAGAC
RT-TP09288-F	CTTGTCGGACGGTATCCCCATT
RT-TP09288-R	CCAAACGGCTTCACGGTTATTA
RT-TP12319-F	TCTTTACCATCGCCGTCCAG
RT-TP12319-R	TGAGAAACACAGTCGGGGCA
RT-TP03955-F	GTGATTGTTTTTGAGCGGGC
RT-TP03955-R	CGAGTCTCCTTTTGGTTGCG
RT-TP04225-F	ACAACAACCACGCCAAACCA
RT-TP04225-R	CGCAACCCACAGAAGCATC
RT-TP09287-F	TCGCTCAGCCGATAACCTTT

RT-TP09287-R	GGGTCATCCTCCCAAGTAATCG
RT-TP04014-F	GGACAGATTTGCCCGAACAG
RT-TP04014-R	TCCAAACAGCAGTGAATCCCA
RT-TP07482-F	TCCACTCAGCAAGCAGCGATA
RT-TP07482-R	AGAGTCACCACCCCCTGTTCAT
RT-TP09267-F	GTGTCGTTATTGCCAGTCCCA
RT-TP09267-R	CTCAGCAAGACCCAAACCACTC
RT-TP09781-F	CCGATGAGAACGGAAAAGCG
RT-TP09781-R	TTCAACGATAGCACTGCCCTC
RT-TPactin-F	TCGCTCTTCCTCACGCTATTT
RT-TPactin-R	GATGTCACGGACGATTTCACG
RT- TP08514-F	CGGGGCTCTTTATCTCTCTGA
RT- TP08514-R	GTTGCCTCGCCATTTGCT
RT- TP09412-F	ATAACACCCACTACCAAATCTTCG
RT- TP09412-R	GCACCGTTCAAACCGCAA
RT-TP05820-F	TACGCAATGTCCTCAAACTCG
RT-TP05820-R	CAATAGACTCAGCAGCGGCAC
**Primers used for probes amplification for EMSA**	
TP04014-L-F	CCACATACCCTTATAAAGCGGA
TP04014-L-R-EMSA	FAM-TAAATGATAGAACTGGTTGAGCGT
TP09267-L-F	CCGAGGCAATCTCCCGTA
TP09267-L-R-EMSA	FAM-GAGGAGAGACGAGAGAAAGTCATAA
TP09412-L-F	GAGATCGAAAGTTTTGCCTTATTAG
TP09412-L-R-EMSA	FAM-TTTGTCGATTGCTTCTGACTGTT
TP08514-L-F	CGAATGGCAACTCTGGCAC
TP08514-L-R-EMSA	TAM-GTCGCCATGTTAGACATCAGTC
TP05820-L-F	GAGCATTTGAAACTCGCAGTG
TP05820-L-R-EMSA	FAM-TCGATATCAAGCACCATGTTGA
β-tubulin-F	ACCTCACTTGCTCCGCTCTG
β-tubulin-R	FAM-ACAAACTTCATAGATGGAGTGGACA
**Primers used for the construction of deletion mutants**	
benA -F	GGAGTAGTTGATTTTCCAGGGAC
benA-R	GGATCCCCGGGGGATC
benA -V-F	TCATTCCACTCAACATTCAGGC
benA -V-R	ACACACAGGAAAACATTGACCG
TP02310-L-F	TGATGTGACCGCCTGGAT
TP02310-L-R	GTCCCTGGAAAATCAACTACTCCCACTCAACGCCAGCAGC
TP02310-R-F	GATCCCCCGGGGATCCATTTAGTGTAGATCATATCCAATCA
TP02310-R-R	CCGAGGTCAACCGCTAC
TP02310-NF	GCAGTGGTGCCTATCCGT
TP02310-NR	GTGGCAATGCTACTGAAAGG

TP02310-F	AATGAATCGCCAGGTAGGA
TP02310-R	GGTCCTCTTCCG
TP06128-L-F	TCGTGTCATCTTCCAGGGTT
TP06128-L-R	GTCCCTGGAAAATCAACTACTCCATTCCAGAGGTCGAGTGAGTA
TP06128-R-F	GATCCCCCGGGGATCCACAACTATCTGTTTGAATATTTGAG
TP06128-R-R	CCAAGAAGCGAAACAGC
TP06128-NF	TCACTGGTAAACAGGGAACG
TP06128-NR	TGGCGAACAGAACAAACAG
TP06128-F	CTGATGCTTCCTGCCGTTAT
TP06128-R	CAAGCGTCAAGCGGTGTA
TP03450-L-F	GCAGATTTCCCATCGCAACC
TP03450-L-R	GTCCCTGGAAAATCAACTACTCCGGTTGTGTTGTTTTCTCCAAGTTG
TP03450-R-F	GATCCCCCGGGGATCCCACTGGAATTGAAAGAGGATA
TP03450-R-R	CCTTTATCGCCTGGGAC
TP03450-NF	GGAGGAGACAGAGCCA
TP03450-NR	GAGCAACGCAATCAAG
TP03450-F	TGAACCCGCTCCCAGAC
TP03450-R	CGCCGTGACTATGCTTT
TP00297-L-F	CTGGGTTGTGGAGGCT
TP00297-L-R	GTCCCTGGAAAATCAACTACTCCTTTATGATCAAGGTCTGCTG
TP00297-R-F	GATCCCCCGGGGATCCGCCATCAGGCAAGAGC
TP00297-R-R	GAATTGGACGGCACCC
TP00297-NF	CTGGGTTGTGGAGGCTTCA
TP00297-NR	CCTTGTTGGAAGAAGCGAAAT
TP00297-F	GCCTGGGATACCTCTGAT
TP00297- R	AATCTCGCCCGCTTAT
TP03988-L-F	ACCCAACCATCCCACAAA
TP03988-L-R	GTCCCTGGAAAATCAACTACTCCAGCGATCACATTCGGATGTAACT
TP03988-R-F	GATCCCCCGGGGATCCCTGTAATTGTTTCTCCGTTTATTTA
TP03988-R-R	CCTTGTGGGTTGTGGTC
TP03988-NF	ACCCAACCATCCCACAAAAC
TP03988-NR	AAGCCGAGCCCAGCAAG
TP03988-F	CGGCGAGGAAGAAGAATC
TP03988-R	CGGCTGCTGTAGAAGACTCC
TP07409-L-F	TAGGGATTGCGAATGGTG

TP07409-L-R	GTCCCTGGAAAATCAACTACTCC CTCAAATCCGATTAACTAGGC
TP07409-R-F	GATCCCCCGGGGATCC GTCATAAATCTTATGAAACATCTTTCC
TP07409-R-R	CAACCCTCACCCTCATCCT
TP07409-NF	ATTGCGAATGGTGGTTTAGAC
TP07409-NR	TGCCCCAAATTCCATAGTG
TP07409-F	GACAGCACCAAACTCACCAC
TP07409-R	GAAGGCAGCGTTGAGGC
TP09544-L-F	TTACTAACCGACGAGCCGC
TP09544-L-R	GTCCCTGGAAAATCAACTACTCC TGGTGGCGGCACCG
TP09544-R-F	GATCCCCCGGGGATCCAATGTCGTGACTCGAG GTGATTATA
TP09544-R-R	CGACGAAGCCGAAGATGACT
TP09544-NF	ATGTTGGCACCATTACG
TP09544-NR	CCCGCCACATAACAGC
TP09544-F	AAATGGTTGGGCGAAGG
TP09544-R	CTCCGTAAGCAATCCAGTAGG
TP08445-L-F	GAGTCCGATTTACCGCTGAT
TP08445-L-R	GTCCCTGGAAAATCAACTACTCCCCTAGAGGCA AGCTTAATAAACTAA
TP08445-R-F	GATCCCCCGGGGATCCCTGTACACCCTCGTGG TTTGC
TP08445-R-R	GGGTTGCCGCAAGGTACA
TP08445-NF	GAGTCCGATTTACCGCTGATAC
TP08445-NR	GGGTTGCCGCAAGGTAC
TP08445-F	AGCAAAGAACAATGACCCTG
TP08445-R	TTAGGCGGTGGGTTTGA
TP08615-L-F	CCGAGTTGACGAGCGAAGC
TP08615-L-R	GTCCCTGGAAAATCAACTACTCCAGGGAGATAT CAGACATCAAGATCA
TP08615-R-F	GATCCCCCGGGGATCCGAGAGTAAGCAATAAG GGTTT
TP08615-R-R	ATCCCAGATGGCTCCC
TP08615-NF	GTGATCGTACCCCGAAGAT
TP08615-NR	CCAGATACATGCGAGGAACA
TP08615-F	AGAAACCGAAAGTTCCCTACA
TP08615-R	GCATCTGCCTGTCTGGT
TP08885-L-F	CACGGTGCCTGGGAGATG
TP08885-L-R	GTCCCTGGAAAATCAACTACTCCGCTCCAAGT GTGCTTCATGTTTG

TP08885-R-F	GATCCCCCGGGGATCCAACTTTATAAAACATGTAATTACTG
TP08885-R-R	CGATGGGCTTGACCTG
TP08885-NF	GTTCCGCGTAGCCCACA
TP08885-NR	TGACTTGCCTCGGGTGG
TP08885-F	CCGAAACCACGCATCT
TP08885-R	GCTTCCCGCAGTACACTA
TP09505-L-F	TGGCTGGCTTGGTTTG
TP09505-L-R	GTCCCTGGAAAATCAACTACTCCGCTTCACGGTTAGTTTCCA
TP09505-R-F	GATCCCCCGGGGATCCATCATGGTTGGAGTCTGATTCAT
TP09505-R-R	CCGCAGCAATGACAAGGT
TP09505-NF	GCTGGGAATCCGGCTCT
TP09505-NR	GACCGTTCAAACACCTTCCA
TP09505-F	CGCTGCCTAAGCCTGAA
TP09505-R	ATGCGAGGAAGGAGGAAA
TP05940-L-F	ATACCCCAGTTACCATACCAGC
TP05940-L-R	GTCCCTGGAAAATCAACTACTCCTTCTTACGTACTAATCTTGCAAGA
TP05940-R-F	GATCCCCCGGGGATCCTCAGTGCGGGTTAATGTCTT
TP05940-R-R	ATGATTTTGTAGCGGTCAGGTT
TP05940-N-F	ACACTTGTCTTGGTTGGGAACATTCTCACG
TP05940-N-R	ACAGCGGAACTTTTTCTCGGGGGAGCAATC
TP05940-F	ATGGACCTCCTTCAGAGTCATA
TP05940-R	TCAAAGTGATAGACTCTCCATCTTA
TP06213-L-F	TTGTTCAGGAATGTATGTGGTCA
TP06213-L-R	GTCCCTGGAAAATCAACTACTCCGGCTAGTTATATATAAATTTCGACA
TP06213-R-F	GATCCCCCGGGGATCCAACTTTCATACATTGAAAAGCCG
TP06213-R-R	GTGATTAGGCGTTCATCGTTAT
TP06213-N-F	AGGCTCTCATACGTCATTTTAGG
TP06213-N-R	CGCTATTGTGAGATGAAAGTGTGGTCGCTG
TP06213-F	ATGCGTCCAACAAATGGAG
TP06213-R	TCATTTCAAACCACTGAATGAAT
TP06945-L-F	AAATCCCAAAGGGTCTTACTGT
TP06945-L-R	GTCCCTGGAAAATCAACTACTCCTTAACTCGGAGAGAACTCCGG
TP06945-R-F	GATCCCCCGGGGATCCTCATCACCGATAATGACAGTATACA
TP06945-R-R	CTCATTCGTTTATGTTCCTTTCG

TP06945-N-F	GTCAGTTCAGACAGCCATTCCGAAGAGGTT
TP06945-N-R	GCCGCAATCTCTCCTTCATCTTCTTCTCCG
TP06945-F	ATGTGCCCCACACCAATA
TP06945-R	TCAATATATGTTGCCCTCGT
TP09568-L-F	GAGATTAGGACATAGAAATGAGCCA
TP09568-L-R	GTCCCTGGAAAATCAACTACTCCTGGACGGCG GCTTGCAG
TP09568-R-F	GATCCCCCGGGGATCCCATTGGAAGACGACGA CA
TP09568-R-R	TTCTTTATTCGGCAACGGTG
TP09568-N-F	GTGTGAATGCTTGACCGATCCTATG
TP09568-N-R	GCACTGTAGTAAGAAGCCGATTAGTCAAGA
TP09568-F	ATGGCTTTCAACGCTACC
TP09568-R	TCAACGAACAGTCCTCACAC
TP02294-L-F	GTGCTCAACTAATTGGTCGGGT
TP02294-L-R	GTCCCTGGAAAATCAACTACTCCTGCGTCTTTT TCTGTTCTCG
TP02294-R-F	GATCCCCCGGGGATCCCCAGCTTACCACTTTTA CTTG
TP02294-R-R	TTCTCACTTTCGCAATACAACATCT
TP02294-N-F	CCGCTGACCAACACCTAAG
TP02294-N-R	AGAGTCCGATGTTATGTTCCTG
TP02294-F	ATGTCTGCCTACACCATGTTAGC
TP02294-R	TCAAACGTTGAGCAAATCTTC
TP02980-L-F	TCCTGGCTCTAAGTTTCCCTGA
TP02980-L-R	GTCCCTGGAAAATCAACTACTCCCTTCGACACA AAAATACGATTTTTC
TP02980-R-F	GATCCCCCGGGGATCCTATTTTTGCTCGTTCAA TTCCTTAT
TP02980-R-R	ATGAGTGAGTGGACCTAAGCAG
TP02980-N-F	CTTCTTTGGTCTATGGATTGCG
TP02980-N-R	CTGTTGAATAATGTTCCTGAAGACTA
TP02980-F	ATGACGATTGCTTCTGTTTC
TP02980-R	TCAATCACGAAAACGCGC
TP04628-L-F	CGAGTGGCAGTAAATAAGTCCCG
TP04628-L-R	GTCCCTGGAAAATCAACTACTCCGTGCTCGTTT GAGTTTCTTCCTG
TP04628-R-F	GATCCCCCGGGGATCCTGGACATAGTGCCATT TGAA
TP04628-R-R	AAAAGTTGCTTGGACCTATTGG
TP04628-N-F	GCTTATCACGGAGCCAGTCT
TP04628-N-R	GTCCCGACACCATTGTTGATAGA
TP04628-F	ATGGCTTTGCGGCGA

TP04628-R	TCATTCAACCCGGTCCAAA
TP04707-L-F	ATACTATCTGCCCTTCCGTCAT
TP04707-L-R	GTCCCTGGAAAATCAACTACTCCCTGCAAGTATTCAGATCTCAACTAA
TP04707-R-F	GATCCCCCGGGGATCCCACTCAACCATGTCAAGTCGTG
TP04707-R-R	CTCCGATTCCTGCTCGTAAGAT
TP04707-N-F	GATTGGATGACAGGATAGGACGAC
TP04707-N-R	TTGGGAGATTACGGGAGATGG
TP04707-F	ATGTCTCGTCAACAAAAGATTCT
TP04707-R	CTATGGGTAACCAAATTGCCC
TP05119-L-F	CAGATTTTGGTCTACGGGGTC
TP05119-L-R	GTCCCTGGAAAATCAACTACTCCATGGTGTCAGTTAATGGGTGGT
TP05119-R-F	GATCCCCCGGGGATCCTGTCTGTGGCGCAACTAAAG
TP05119-R-R	CAACGGCTAATCTAAATCTCGCT
TP05119-N-F	GACATTCCTGGTATCCTTGCTCA
TP05119-N-R	CGTTACATAAGGCATATTCAGGGA
TP05119-F	ATGAAACGCACTTCCCAA
TP05119-R	CTAAACTAATAACTCAATATCTTTG
TP05236-L-F	GAACCAGGTGAACAAAGTCAAG
TP05236-L-R	GTCCCTGGAAAATCAACTACTCCTTTGCAAAATCACCCCAGAG
TP05236-R-F	GATCCCCCGGGGATCCCCAGCCAGCGCATAACTC
TP05236-R-R	CGGAATCGCCTCACGAAC
TP05236-N-F	ACAAAAGGCTGCTACTGAAACG
TP05236-N-R	CCACCTGGGCACTGAATAAA
TP05236-F	ATGGGAAATATAGGAGGACCAAG
TP05236-R	CTATTCGTCCGGTATATCCACATT
TP05290-L-F	TTACATCTCCCCTTCCATTCG
TP05290-L-R	GTCCCTGGAAAATCAACTACTCCAATTAATGCATTAATTTGTTTTGTG
TP05290-R-F	GATCCCCCGGGGATCCCTGTGGATCACAAAACATCTATCA
TP05290-R-R	TCGTTCTGATGATAGGACAAGC
TP05290-N-F	CCTAGTTGGAAGCATTATA
TP05290-N-R	AGATCACAGTTCGATTCTTAGTTT
TP05290-F	ATGCCGAGAGACAGGGAATC
TP05290-R	TTAAACCTCCTCGAAGGCAGT
TP05746-L-F	TACGATTCCTTGATTCCCACAT

TP05746-L-R	GTCCCTGGAAAATCAACTACTCCTCTCGGGCT GCTGTACTAAC
TP05746-R-F	GATCCCCCGGGGATCCAGAGATGAACTGTACG GAGCG
TP05746-R-R	AGACTTCATTTTCTGTTGACTTTG
TP05746-N-F	CCGATAAGATGGACACTGGACA
TP05746-N-R	AGGCGGACCGTCAATCTATG
TP05746-F	ATGAGCCTCACGTACTCACGAT
TP05746-R	TTACGCCGACTGAGGATCATAC
TP06038-L-F	ACGGGCTTCAAGTCACCGATAA
TP06038-L-R	GTCCCTGGAAAATCAACTACTCCCTTCGCTCTA GGTAACGATAATGT
TP06038-R-F	GATCCCCCGGGGATCCCGTGCTATTCGATCATA ATCAAGA
TP06038-R-R	GCAGGTATGAGGATAAACACGC
TP06038-N-F	CGTGATGGGATATTATTGTGCGATACTAA
TP06038-N-R	CGATTTAGAATAGTTTCACACATCAGAGT
TP06038-F	ATGGCTGCACTCCGTGA
TP06038-R	TCATGAAGCATCAAGTAAATCC
TP06973-L-F	GTTTGGCAGTCTGGTCGGTCT
TP06973-L-R	GTCCCTGGAAAATCAACTACTCCGTTGTGTGGA TATTTTGCTTTT
TP06973-R-F	GATCCCCCGGGGATCCTACACGACCAATAACTA GAAGTTCT
TP06973-R-R	GACGCAAAACTGCCATACTTC
TP06973-N-F	TTCATCACGAACCGCAC
TP06973-N-R	CATATCCGCTGATAAAGGG
TP06973-F	ATGACTGCGGCACGGAATG
TP06973-R	TTATTGCGAACTCAAATTGGG
TP09107-L-F	TTAGGGGCTCCTTTATGTGTTG
TP09107-L-R	GTCCCTGGAAAATCAACTACTCCCTTCGATGTG GCGATTTTGA
TP09107-R-F	GATCCCCCGGGGATCCGCATTCCTACTGGCAA CACTG
TP09107-R-R	CATAAACTCCAAACAGACCAAGC
TP09107-N-F	AACTTGTTTGACTTAGCCGA
TP09107-N-R	AACCAACCTGACACTCCATT
TP09107-F	ATGCCGAAGGCAAAACAA
TP09107-R	CTAGACATGATTATGGTTGAACATA
TP09510-L-F	TTTGCGGATATTAGCAGGTG
TP09510-L-R	GTCCCTGGAAAATCAACTACTCCGGGTGCGAG GATTTACTTTT

TP09510-R-F	GATCCCCCGGGGATCCTCTTCCTTCTCGTTTAG GT
TP09510-R-R	CAAAGTATTTAGAAAGGCGT
TP09510-N-F	AAACAAAAATCATCCAAAGCAG
TP09510-N-R	GGATGTTGGCTCGGCAG
TP09510-F	ATGGCTTATGAAACTCTCTC
TP09510-R	TTATCGAGCCCTTTCAG
TP12095-L-F	TTGTGTTACCAATCCATCGTTAC
TP12095-L-R	GTCCCTGGAAAATCAACTACTCCTTCGACGCTG AGCGCTC
TP12095-R-F	GATCCCCCGGGGATCCGCGTGTGTTTTCAATT CCG
TP12095-R-R	CAGGGGGAAACTTACTTGGC
TP12095-N-F	CATAATAGAAAACGAACCAAACCAG
TP12095-N-R	CATCGCCCTTCAGCAGACC
TP12095-F	ATGTCTCGGCGACTCTCGT
TP12095-R	TCAAATAATGATTGACTGTTGCTG
TP09286-L-F	GTCTTGACGAGTCTATGGAGCAC
TP09286-L-R	GTCCCTGGAAAATCAACTACTCCTTTTAAGATG TTAAGCAGTTTGCA
TP09286-R-F	GATCCCCCGGGGATCCCCATTTTAAACGACGA CATGG
TP09286-R-R	CCTTAGCCGCACTGAACCTG
TP09286-N-F	TCGTCGTCTTTCCCAACTGG
TP09286-N-R	CATCCCTCCAGCACCTCCTT
TP09286-F	ATGACTTCCATTTTCAAGTCCGA
TP09286-R	TCATTGCGGAAAGAATTGATTAGAA
TP09590-L-F	GCCTAAAGAGCATGGACGG
TP09590-L-R	GTCCCTGGAAAATCAACTACTCCTACATGTGGC CCAAACGG
TP09590-R-F	GATCCCCCGGGGATCCATACCAGGTTCTTTTGA GT
TP09590-R-R	CCAATTCAACACTGCCACCT
TP09590-N-F	GGAGTACCGGAATCAGAAAGG
TP09590-N-R	CAAACGTGCCGCCATTAC
TP09590-F	ATGTCCGGAAGAAACGCTC
TP09590-R	CTAAAACACCCTAACGCCCAT
**Primers used for the complementation**	
CTP06128-L-F	TCAAATCAAAGCGTCCAAC
CTP06128-R	CGACCGTTATATAGTCAAGCAATCAGGCGACAA CAACCACG
TP06064ter-F	TTGCTTGACTATATAACGGTCG
TP06064ter-R	CGTCGTAGAAGCGGTGGT

Ble-F	ACCACCGCTTCTACGACGCCCACACACCATAG CTTCA
Ble-R	AGCTTGCAAATTAAAGCCTT
CTP06128-R-F	AAGGCTTTAATTTGCAAGCTACAACTATCTGTTT GAATATTTGAG
CTP06128-R-R	CACCACGTCACCCTGATC
CTP06128 -NF	GGTTTCACTGGTAAACAGGGA
CTP06128 -NR	GAGGCGCAAACTAGATTCTGA
**Primers used for probe amplification for Southern hybridization**	
Probe-F	TTCCGCCTACACTTACTATTG
Probe-R	TCCTACATACGCCGAGCC
**Primers used for DNA binding domain-encoding DNA fragment amplification**	
TP06128-domain-F	GTAGAATTCAAGAAACGAGGTTCCAGC
TP06128-domain-R	GTCAAGCTTTTACTTGGGCGTAAAAGAAGTTGT
